# A history of childhood maltreatment is associated with altered DNA methylation levels of DNA methyltransferase 1 in maternal but not neonatal mononuclear immune cells

**DOI:** 10.3389/fpsyt.2022.945343

**Published:** 2022-11-10

**Authors:** Rezan Nehir Mavioglu, Laura Ramo-Fernandez, Anja M. Gumpp, Iris-Tatjana Kolassa, Alexander Karabatsiakis

**Affiliations:** ^1^Department of Clinical and Biological Psychology, Institute of Psychology and Education, Ulm University, Ulm, Germany; ^2^Department of Psychology, Clinical Psychology II, University of Innsbruck, Innsbruck, Austria

**Keywords:** childhood maltreatment, early life adversity, DNA methylation, intergenerational transmission, *DNMT1*

## Abstract

Childhood maltreatment (CM) is associated with alterations in DNA methylation (DNAm) especially in stress response genes. Due to the higher risk of overall health complications of individuals with a parental history of CM, intergenerational transmission of CM-associated DNAm changes has been investigated but remains unclear. In this study, we investigated if different severities of CM have any influence on the DNAm of DNA methyltransferase 1 (*DNMT1*), an important enzyme of the DNAm machinery, in immune and buccal cells of mother-newborn dyads. DNAm was assessed by mass spectrometry using immune cell DNA from mothers (*N* = 117) and their newborns (*N* = 113), and buccal cell DNA of mother-newborn dyads (*N* = 68 each). Mothers with a history of CM had lower mean methylation of *DNMT1* in immune cells compared to the mothers without a CM history. CM status only influenced maternal *DNMT1* gene expression when at least moderate CM was reported. Buccal cell DNAm was not associated with CM status. Maternal history of CM was not linked to any alterations in *DNMT1* mean DNAm in any of the cell types studied in newborns. We conclude that the CM-associated alterations in *DNMT1* DNAm might point to allostatic load and can be physiologically relevant, especially in individuals with more severe CM experiences, resulting in an activated DNA methylation machinery that might influence stress response genes. Our lack of significant findings in buccal cells shows the tissue-specific effects of CM on DNAm. In our sample with low to moderate maternal CM history, there was no intergenerational transmission of *DNMT1* DNAm in newborns.

## Introduction

Childhood maltreatment (CM) is a severe form of early life stressor and a widespread problem around the world (1) that may lead to physical and mental health problems throughout one’s lifespan (2, 3). One way CM induces such health effects a long time after its exposure might be through epigenetic alterations. Epigenetic alterations are tissue specific and can alter gene expression. One of the epigenetic mechanisms, DNA methylation (DNAm), is known to “shut down” genes when present in promoter regions, by preventing transcription factor binding and reducing gene expression (4). Psychoneuroimmunology research provided evidence that DNAm is affected by CM (5, 6). Epigenetic alterations were mainly reported in genes of the neuroendocrine stress-response such as *NR3C1* and *FKBP5*, indicating mostly a positive CM-DNAm association in *NR3C1* and a negative one in *FKBP5* (5–7). These studies almost exclusively focused on a single tissue, and comparisons between the most two prominently studied tissues, peripheral blood mononuclear cells (PBMC) and buccal cells, are not available. Moreover, CM associated DNAm alterations were also observed in epigenome-wide association studies and studies concerning global DNAm (5, 6), hinting toward the involvement of fundamental regulators of DNAm in the relationship between CM and DNAm outcomes. How DNAm regulators moderate the effects of CM on DNAm outcomes might be through stress-response elements themselves. It has been proposed that glucocorticoid receptor (encoded by *NR3C1*)—glucocorticoid complexes can (1) bind to glucocorticoid response elements (GREs) within genes coding DNA methyltransferases (*DNMTs*) and change their expression (8, 9), or (2) activate specific genes that impact the expression of DNMT enzymes (10). The link between DNMTs and stress-response elements is possibly bidirectional since DNMTs can regulate methylation of the genes coding for these elements.

DNA methyltransferase 1 (*DNMT1*) is one of the five DNMT enzymes that are responsible for the transfer of methyl groups, along with DNMT2, DNMT3A, DNMT3B, and DNMT3L. DNMT1, DNMT3A, and DNMT3B are known to be canonical cytosine 5-DNMTs that catalyze the addition of methyl (–CH_3_) groups to cytosine of CpG dinucleotides (CpG sites) in both strands of genomic DNA (11). DNMT1 is both a maintenance and *de novo* methyltransferase meaning that it is involved in the methylation of hemimethylated CpG sites and contributes to the methylation of new CpG sites (12). Because of its crucial role in DNAm regulation, DNMT1 has been studied in the context of early life adversity and psychopathology in both animals and humans. In rats, increased DNMT1 mRNA levels in fetal cortex after prenatal stress was reported (13). Similarly, in male mice, increased DNMT1 protein levels were found in the frontal cortex after prenatal restraint stress (14). Another study showed the long-term effect of a maternal separation paradigm in male mice, reporting increased DNMT1 mRNA in the prefrontal cortex (PFC) in adult animals but not in 15 days-old animals (15). However, Blaze and Roth (16) reported less DNMT1 mRNA in PFC of adult male rats after experimental CM exposure. Overall, the results of these animal studies indicate that DNAm machinery is affected by early-life adversity on the levels of gene and protein expression. There have been no studies so far concerning the influence of early life adversity or CM on *DNMT1* DNAm or gene expression in humans. Instead, the scientific focus was mostly directed on lifetime trauma and psychopathology. A longitudinal study reported increased levels of DNAm of one CpG site in *DNMT1* in the venous blood of individuals with PTSD after trauma exposure, compared to their pre-trauma DNAm levels (17). However, there were no significant differences in DNAm after trauma in PTSD cases versus controls. Another study with mother-newborn dyads found a positive association between one methylation cluster of *DNMT1* and genome-wide mean DNAm only in maternal venous blood but not in newborn cord blood (18). In addition, a negative association was found between war trauma and maternal genome-wide mean DNAm in their cohort with a history of stressful life events. The association between war trauma and *DNMT1* DNAm was not directly tested (18) but results from these two studies hint toward an adulthood trauma-related regulation of *DNMT1* DNAm in blood. *DNMT1* gene expression levels were also not reported in these two studies (17, 18), limiting the evaluation of possible physiological effects of altered DNMT1 methylation. As for studies investigating the role of *DNMT1* in psychopathology, a postmortem design with individuals with schizophrenia and bipolar disorder reported increased DNMT1 expression and enzymatic activity patients’ brains, compared to controls (19). They reported increased DNMT1 binding to GABAergic and glutamatergic promoters that led to the downregulation of these genes. This downregulation did not necessarily correlate with methylation of GABAergic and glutamatergic promoters, hinting toward other crucial roles of the DNMT1 enzyme beyond DNAm (19, 20). In line with this finding, antidepressant medications might reduce DNMT1 activity, which might be one of the mechanisms how these medications work in treating individuals with various mental health disorders (21, 22). This view was supported by a conditional forebrain DNMT1 knockout mice model showing less depressive-like behavior compared to wild-type mice (23). These studies show the importance of DNMT1 in the context of lifetime adversity and psychopathology.

As a cumulative stress factor, maternal history of CM might also influence next generations’ health. The offspring of individuals with a history of CM were shown to have a higher risk of developing physical as well as mental disorders (24, 25). One of the pathways that can explain this observation could be the intergenerational transmission of epigenetic stress profiles. There are two main ways how intergenerational transmission of trauma might occur in DNAm context. Firstly, intergenerational transmission of CM-related DNAm changes might refer to a direct transmission of DNAm signatures from parental gametes. The majority of methyl marks on the DNA are thought to be removed and not passed to the further generations, thus intergenerational transmission of DNAm implies incomplete removal of the methyl groups. There is no evidence so far that this form of intergenerational transmission of trauma exists in the DNAm of humans but it has been observed in some animals, limited to only a certain number of generations (26). However, the cross-species comparability of methylation regulation processes remains under debate. Another form of intergenerational transmission of trauma is through alterations in maternal physiology and increased prenatal stress that can affect the intrauterine development and the epigenetic makeup of the fetus (27). In extreme prenatal stress conditions, some studies conducted with animals (28, 29) as well as humans (30, 31) reported findings indicating intergenerational transmission of DNAm markers. Intergenerational transmission studies that are conducted later on in human life have the limitation that they can be confounded by certain environmental factors such as parenting style, shared lifestyle factors and other psychosocial influences. Although parenting behavior can be affected by a history of traumatic events, they can only qualify as “indirect” intergenerational transmission, since the impact occurs after birth and does not directly affect the gestational biology (27). Therefore, it is essentially important to study the intergenerational transmission of DNAm directly after birth. In our cohort of mother-newborn dyads, we did not find evidence for a transmission of maternal CM-induced DNAm in stress-response related genes in immune cells collected after birth of the newborn (7).

In this study, we aim to understand if a history of CM experiences can influence the DNAm profiles and gene expression of an important regulator of DNAm, DNMT1. To achieve this, we examined the role of different severities of CM on *DNMT1* DNAm in PBMC and buccal cells of mothers who recently gave birth. *DNMT1* relative gene expression levels were determined to assess the possible physiological implications of alterations in DNAm. To test if stress-response elements have any bidirectional relationships with DNMTs as proposed before (8–10), the associations between our previously published *NR3C1* exon 1F DNAm and relative gene expression results from the same cohort (7), and *DNMT1* DNAm and relative gene expression were explored in maternal PBMC. Lastly, we examined maternal CM related intergenerational *DNMT1* DNAm transmission in newborn PBMC from umbilical cord blood and buccal cells both collected also shortly after parturition.

## Materials and methods

### Participants

As part of the “My Childhood—Your Childhood” study, *N* = 548 clinically healthy mothers were recruited between October 2013 and December 2015, within the first week after giving birth (*M* = 2.71 days) at the Department of Obstetrics and Gynecology at Ulm University Hospital. Mothers were asked to provide written informed consent forms and answer a set of sociodemographic and psychological questionnaires. Peripheral blood of consenting mothers, umbilical cord blood of their newborns, and cheek swabs of mother-newborn dyads were collected. Out of 153 mothers that provided blood, only 58 mothers had a history of at least low CM. For the epigenetic analyses of PBMC, these mothers were matched with *N* = 59 mothers without a history of CM, resulting in a cohort of 117 mother-newborn dyads. Four newborn samples were excluded from the analyses (*N* = 3 twins, *N* = 1 sample loss due to technical difficulties during the cell isolation procedure), leaving *N* = 117 mothers and *N* = 113 newborns in the immune cell cohort [please see Ramo-Fernández et al. (7) for details about the selection procedure]. From these participants, a subsample of 68 mothers and infants was defined according to maternal CM status and availability of at least 1 μg DNA extracted from buccal cells for measurements of buccal cell epigenetics (buccal cell cohort). The study protocol was in accordance with the Declaration of Helsinki, which was approved by the ethics committee of Ulm University.

### Sociodemographic and psychological measures

Mothers provided sociodemographic and health information, such as age, newborns’ sex, smoking, and medical history. Please find the information about the relevant variables in Table 1.

**TABLE 1:** Sociodemographic and health information in mother-newborn dyads in immune and buccal cell cohorts, categorized according to low and moderate childhood maltreatment (CM) severity groups.

		CM_*low*–_	CM_*low+*_	CM_*mod*–_	CM_*mod+*_
		*N* = 59	*N* = 58	*N* = 84	*N* = 33
Immune cell cohort (*N* = 117)	Maternal age (Mean ± SD, years)	33.08 ± 4.23	32.79 ± 4.55	33.01 ± 4.22	32.76 ± 4.80
	CTQ sum score (Mean ± SD)	27.12 ± 1.89	40.17 ± 12.13	29.18 ± 4.34	44.82 ± 13.95
	Infant sex [male *N* (%)]	35 (59.3)	30 (51.7)	49 (58.3)	16 (48.5)
	Smoking during pregnancy [yes *N* (%)]	5 (8.5)	6 (10.3)	6 (7.1)	5 (15.2)
	Chronic illness [yes *N* (%)]	18 (30.5)	24 (41.4)	29 (34.5)	13 (39.4)
	Lifetime psychiatric diagnosis [yes *N* (%)]	12 (20.3)	17 (29.3)	19 (22.6)	10 (30.3)
	Maternal lymphocyte to monocyte ratio (Mean ± SD)	3.42 ± 1.17	3.3 ± 1.18	3.36 ± 1.25	3.39 ± 0.97
	Newborn lymphocyte to monocyte ratio (Mean ± SD)	3.89 ± 2.03	3.94 ± 3.66	4.13 ± 3.13	3.26 ± 1.66

		***N*** **= 34**	***N*** **= 34**	***N*** **= 45**	***N*** **= 23**

Buccal cell cohort (*N* = 68)	Maternal age (Mean ± SD, years)	33.35 ± 4.00	32.21 ± 5.01	33.29 ± 4.05	32.76 ± 4.80
	CTQ sum score (Mean ± SD)	27.35 ± 1.91	41.71 ± 13.41	29.47 ± 4.97	44.43 ± 15.16
	Infant sex [male *N* (%)]	19 (55.9)	17 (50.0)	25 (44.4)	12 (52.2)
	Smoking during pregnancy [yes *N* (%)]	5 (14.7)	6 (17.6)	6 (13.3)	5 (21.7)
	Chronic illness [yes *N* (%)]	13 (38.2)	13 (38.2)	16 (35.6)	10 (43.5)
	Lifetime psychiatric diagnosis [yes *N* (%)]	9 (26.5)	11 (32.4)	13 (28.9)	7 (30.4)

To assess the history of CM, the German short version of the *Childhood Trauma Questionnaire* (CTQ) (32, 33) was filled in by the mothers. The CTQ is a Likert-type questionnaire with five subscales (emotional abuse, physical abuse, sexual abuse, emotional neglect, and physical neglect), scores of each can range from 5 to 25, with higher scores indicating a more severe history of CM. The CTQ sum score is calculated by adding each subscale score together, and the total score can range from 25 to 125 to measure CM load. CTQ was used to dichotomize the mothers according to their low and moderate CM experiences. To achieve this, mothers were categorized as being experienced none to mild, low, moderate or severe maltreatment in each of the five subscales, according to the CTQ manual (33). For low dichotomization, mothers who experienced low to severe maltreatment according to at least one subscale were classified as CM_*low*+_. The mothers who did not fulfill this criterion were classified as CM_*low*–_. Similarly, for moderate dichotomization, mothers who experienced moderate to severe maltreatment according to at least one subscale were classified as CM_*mod+*_. The mothers who did not fulfill this criterion were classified as CM_*mod*–_.

### Sampling of immune cells and buccal cells for DNA isolation

Peripheral blood mononuclear cells (PBMC) and umbilical cord blood mononuclear cells (UBMC) were isolated by Ficoll-Hypaque density gradient centrifugation (GE Healthcare, Chalfont St Giles, UK) according to the manufacturer’s protocol, from maternal peripheral blood and newborn umbilical cord blood that were collected into CPDA-buffered blood collection tubes (Sarstedt S-Monovette, Nürmbrecht, Germany). Cells were cryopreserved in medium (1:10 dimethyl sulphoxide: fetal calf serum: Sigma-Aldrich, St. Louis, MO, USA) and stored at –80°C until DNA isolation. Additionally, a K3EDTA-buffered collection tube was used for samples from mothers and newborns and handed to the Department of Clinical Chemistry and Central Laboratory of Ulm University for standard hemogram analysis. Buccal cells (BC) were collected from mothers and newborns with buccal swabs (Isohelix, Harrietsham, Kent, United Kingdom), and the swabs were placed at –80°C until DNA isolation.

DNA was purified from PBMC, UBMC and BC using an automated MagNAPure 96 platform (Roche, Basel, Switzerland). DNA concentrations were measured with a Qubit fluorometer (Life Technologies, Carlsbad, CA, USA) and DNA eluates were set to 40 ng/μl. DNA samples were stored at –20°C and sent to Varionostic GmbH (Ulm, Germany) for DNAm analyses.

### DNA methylation analyses

Thawed DNA samples (1 μg minimum) were treated with bisulfite, amplified with two primer pairs resulting in two amplicons by polymerase chain reaction (PCR), reverse transcribed and treated with RNase A, before performing EpiTYPER mass spectrometry (Agena Bioscience, San Diego, CA, USA). RNase A cleaves the reverse transcribed PCR products at the uracil residues, therefore the resulting products consist of CpG units that can contain multiple CpG sites. Products with too high or too low mass cannot be successfully read, as a limitation of the technique (34). The DNA methylation percentage of a CpG unit represents the average DNA methylation percentage of the CpG sites in that unit. The primers were chosen to span the regulatory regions in the first intron of the *DNMT1* gene [Chr19:10,194,327-10,194,571 (GRCh38/hg38), primer sequences P_*F*1_: 5′-AGGAAGAGAGGATGTATAGTTTTGGGGGAAAGGTA-3′, P_*R*1_: 5′-CAGTAATACGACTCACTATAGGGAGAAGGCTAAAAATTCTCTTTTAATCCCCAAAT-3′], and the beginning of the first exon including the gene promoter [chr19:10,194,533-10,195,072 (GRCh38/hg38), primer sequences P_*F*2_: 5′-AGGAAGAGAGTTAAAGTTTGTTGTATTTGGGGATT-3′, P_*R*2_: 5′-CAGTAATACGACTCACTATAGGGAGAAGGCTTTCCATCCTTCTACACAAAATATC-3′]. The chosen sequence had an overlapping length of 746 bp with a total of 73 CpG sites, and 69% GC content. Please see Supplementary Figure 1 for a detailed depiction of the sequence, as well as regulatory regions, and the CpG sites. Along with the promoter, the sequence contains enhancers, microRNA targets, and transcription start sites (TSS). Please see Supplementary Table 1 for the genomic and regulatory regions in the sequence, their positions, and the corresponding CpG units.

*NR3C1* exon 1F methylation assessment was performed as a part of a previous study (7). Briefly, primers were chosen to span *NR3C1* exon 1F, to assess the sequence Chr5:143,413,957-143,414,659 (GRCh38/hg38). The chosen sequence had a length of 703 bp with a total of 79 CpG sites. Please see Ramo-Fernández et al. (7) for details.

### Gene expression analysis

Gene expression analyses were performed in *N* = 72 mothers due to the limited amount of remaining PBMC. Qiagen RNeasy Kit (QIAGEN, Hilden, Germany) was used for RNA isolation. After quantification with a Qubit spectrophotometer (Life Technologies, Carlsbad, CA, USA), RNA was transcribed to cDNA using a high-capacity cDNA reverse transcription kit (Thermo Fischer Scientific, Waltham, MA, USA). Please check Ramo-Fernández et al. (7) for details about RNA purification and cDNA conversion. Gene expression assays (real-time quantitative PCR) were performed using QuantStudio 6 (Applied Biosystems, Waltham, MA, USA) with TaqMan Gene Expression assays (Thermo Fischer Scientific, Waltham, MA, USA) for *DNMT1* as the target gene (Hs00154749_m1; covers exon boundary 1-2) and *IPO8* as the housekeeping gene (Hs00183533_m1). Each cDNA sample (20 ng) was measured in triplicates and an interplate calibrator which was prepared as a mixture of the sample cDNA was used to control for interplate differences in a total of six plates. Relative DNMT1 gene expression of the samples was calculated using the Livak method, with the interplate calibrator as the reference sample [2^–ΔΔ^*^Ct^*, Livak and Schmittgen (35)].

*NR3C1* relative gene expression was performed for another study (7). Briefly, with the same QuantStudio 6 (Applied Biosystems, Waltham, MA, USA), a real-time quantitative PCR was performed using TaqMan Gene Expression assays (Thermo Fischer Scientific, Waltham, MA, USA) for *NR3C1* as the target gene (Hs00353740_m1; covers exon boundary 4-5) and *SDHA* (Hs00188166_m1) and *IPO8* (Hs00183533_m1) as housekeeping genes. Please see Ramo-Fernández et al. (7) for details.

### Data processing and statistical analyses

Raw methylation data with the mean percentages of each CpG unit were pre-processed to verify high data quality. In summary, CpG units unable to be analyzed in more than 30% of the whole sample, as well as samples that had more than 50% of missing values overall, were excluded from the analysis. 32 CpG units (57 CpG sites), samples from *N* = 109 mothers and *N* = 110 newborns for analyses with PBMC (*N* = 102 dyads with both mother and infant data), and *N* = 62 mothers and *N* = 65 newborns for analyses with BC (*N* = 59 dyads with both mother and infant data) remained after this process. The CpG units with the same calculated molecular weight (due to their shared nucleotide content) were grouped together, in the cases of unsuccessful discrimination of the CpG units by mass spectrometry as a limitation of the technique. Mean percentage methylation scores were created by calculating the mean of the available CpG unit methylation percentages. As for *NR3C1* exon 1F mean DNAm, data from *N* = 113 mothers was used and 17 CpG units (29 CpG sites) remained after pre-processing [please see Ramo-Fernández et al. (7) for details].

Due to non-normal distributions and unequal variances, non-parametric tests were performed. Spearman’s rank-order correlation was used to determine the bidirectional relationships between the variables. Wilcoxon signed-rank test was performed for within-subjects analyses, to compare immune and buccal cell DNAm of the same individuals. To compare the DNAm and relative gene expression values of CM− and CM+ groups, Brunner Munzel test (36) with t-approximation was used using rankFD package (37) in R 4.1.3. Briefly, Brunner Munzel test is a non-parametric rank test that can be used when two populations have unequal variances, unlike Wilcoxon tests. Its test statistic is *T*. It measures stochastic equality of two populations, meaning the frequency of larger values is equal among populations. The relative estimate (P) is between 0 and 1, and if it is around 0.5 stochastic equality is given. If it is significantly smaller than 0.5, the first group has a higher frequency of larger values compared to the second group and if it is larger than 0.5, the second group has a higher frequency of larger values (36). Relative estimates were reported with 95% confidence intervals (CI) in brackets such as [Lower CI, Upper CI]. Calculations were performed with CM_*low*–_ or CM_*mod*–_ as the first group. False discovery rate (FDR) correction due to multiple testing was performed according to Benjamini-Hochberg procedure (38). In order to test the effects of possible covariates in the relationship between CM and DNAm or relative gene expression, robust regressions were performed using MASS package (39) in R 4.1.3. These covariates were maternal age, number of days between parturition and blood sampling, history of psychiatric diagnosis, medication use, folic acid intake during pregnancy (*N* = 8 reported), smoking, presence of chronic illness, and lymphocyte to monocyte ratio (for PBMC only) for analyses with mothers. For analyses with newborns, the covariates were gestational age, birth weight, sex, lymphocyte to monocyte ratio (for UBMC only), and the maternal covariates mentioned above. The covariates were tested one by one in separate models. Mean and standard deviations were reported as descriptives for comparability reasons. All tests were performed two-tailed, and alpha was set to 0.05.

## Results

### Descriptives

Please see Table 1 for descriptives for sociodemographic and health variables, categorized by CM status in immune cell and buccal cell cohorts. The comparisons of the variables within CM_*low*_ and CM_*mod*_ groups did not reveal any differences (*p*s > 0.05) except for the CTQ sum scores which differed between CM_*low*–_ and CM_*low+*_ (*T* = 36.496, *P* = 0.970, *p* < 0.001), and CM_*mod*–_ and CM_*mod+*_ (*T* = 16.621, *P* = 0.924, *p* < 0.001) in immune cell cohort, meaning higher CTQ scores in groups with at least low and at least moderate CM history. Analyses with the buccal cell cohort yielded similar results.

### Maternal DNA methylation and gene expression of DNA methyltransferase 1 according to childhood maltreatment status

#### Maternal DNA methyltransferase 1 methylation in peripheral blood mononuclear cells

**FIGURE 1:**
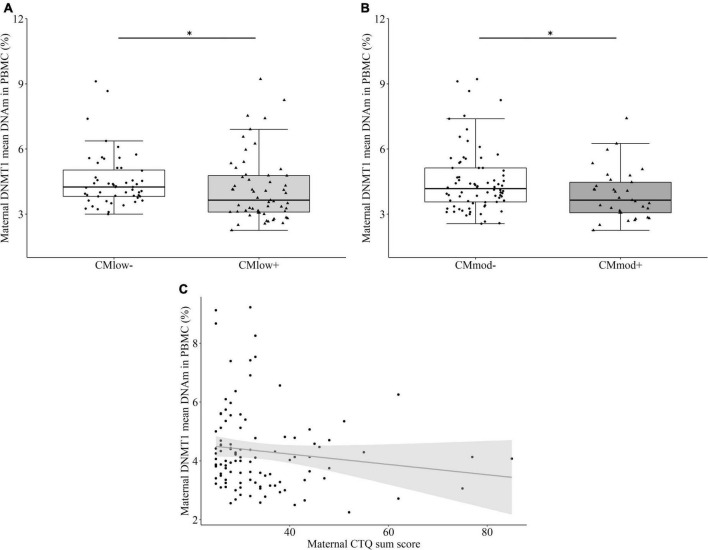
DNA methyltransferase 1 (*DNMT1*) mean DNA methylation (DNAm) and relative gene expression in peripheral blood mononuclear cells (PBMC) of the mothers. **(A)** Mothers with none to mild CM experiences (CM_*low*–_) had higher *DNMT1* mean DNAm compared to mothers with at least low CM experiences (CM_*low+*_; *p* = 0.013). **(B)** Mothers with none to low CM experiences (CM_*mod*–_) had higher *DNMT1* mean DNAm compared to mothers with at least moderate CM experiences (CM_*mod+*_; *p* = 0.040). **(C)** *DNMT1* mean DNAm had a negative trend level correlation with Childhood Trauma Questionnaire (CTQ) sum score (*r*_*s*_ = –0.167, *p* = 0.083). *p < 0.05.

CM_*low+*_ mothers (4.15 ± 1.53%) had lower mean percentage *DNMT1* DNAm than CM_*low*–_ mothers (4.54 ± 1.25%, *T* = –2.527, *P* = 0.363 [0.255, 0.471], *p* = 0.013, Figure 1A). Similarly, CM_*mod+*_ mothers (3.91 ± 1.16%) had lower mean percentage *DNMT1* methylation than CM_*mod–*_ mothers (4.52 ± 1.48%, *T* = –2.108, *P* = 0.373 [0.253, 0.494], *p* = 0.040, Figure 1B). These findings did not change when tested with the covariates (see Section “Data processing and statistical analyses”). Mean percentage *DNMT1* methylation had a trend level negative association with CTQ sum score (*r*_*s*_ = –0.167, *p* = 0.083, Figure 1C). Analyses with single CpG units revealed significant differences depending on CM status with overlaps in low and moderate cut-offs that survived FDR correction (see Supplementary Tables 2, 3). Twelve out of 32 CpG units differed in terms of mean percentage DNAm of CM_*low*–_ and CM_*low+*_ mothers (Supplementary Table 2), whereas, only two out of 32 CpG units were differentially methylated in terms of CM_*mod*_ status of mothers (Supplementary Table 3). For the CM_*low*_ categorization, differentially methylated CpG units were predicted to be located at enhancers, exon, and TSS (see Supplementary Table 1 for locations of regulatory regions and corresponding CpG units). Except for 2 CpG units, CM_*low+*_ mothers had lower mean percentage DNAm than CM_*low*–_ mothers. Mean percentage DNAm differences ranged from 1.0 to 4.9% (Supplementary Table 2). Two CpG units were detected to be lower in terms of mean percentage DNAm in CM_*mod+*_ compared to CM_*mod*–_ mothers. These units were predicted to be located in the enhancer and TSS. Mean percentage DNAm differences were 1.8 (enhancer) and 7.3% (TSS; Supplementary Table 3).

#### Maternal DNA methyltransferase 1 gene expression in peripheral blood mononuclear cells

**FIGURE 2:**
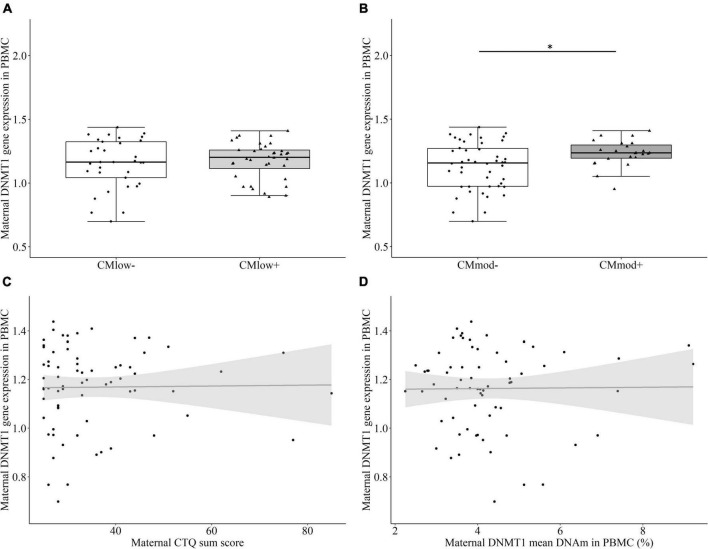
DNA methyltransferase 1 (*DNMT1*) relative gene expression in peripheral blood mononuclear cells (PBMC) of the mothers. **(A)** There was no significant difference between CM_*low*–_ and CM_*low+*_ mothers in terms of their *DNMT1* relative gene expression levels. **(B)** CM_*mod*–_ mothers had lower *DNMT1* relative gene expression compared to CM_*mod+*_ mothers (*p* = 0.015). **(C)** *DNMT1* relative gene expression did not have a significant correlation with CTQ sum score (*r*_*s*_ = 0.034, *p* = 0.782). **(D)** *DNMT1* relative gene expression did not have a significant correlation with *DNMT1* mean DNAm (*r*_*s*_ = –0.012, *p* = 0.921). *p < 0.05.

There was no significant difference in the relative *DNMT1* gene expression between CM_*low*–_ (1.16 ± 1.93) and CM_*low+*_ mothers (1.17 ± 1.44, *T* = 0.151, *P* = 0.511 [0.366, 0.656], *p* = 0.881, Figure 2A). However CM_*mod+*_ mothers (1.23 ± 1.03) had higher relative *DNMT1* gene expression than CM_*mod–*_ mothers (1.14 ± 1.86, *T* = 2.487, *P* = 0.664 [0.532, 0.795], *p* = 0.015, Figure 2B). Therefore, relative gene expression of *DNMT1* differed depending on the severity of CM categorization. The results did not change when certain variables were accounted for as covariates (see Section “Data processing and statistical analyses”). Relative *DNMT1* gene expression did not have an association with CTQ sum score (*r*_*s*_ = 0.034, *p* = 0.782, Figure 2C). No association between mean percentage *DNMT1* DNAm and relative *DNMT1* gene expression was found (*r*_*s*_ = –0.012, *p* = 0.921, Figure 2D).

#### The association between DNA methyltransferase 1 and *NR3C1* in peripheral blood mononuclear cells

**FIGURE 3:**
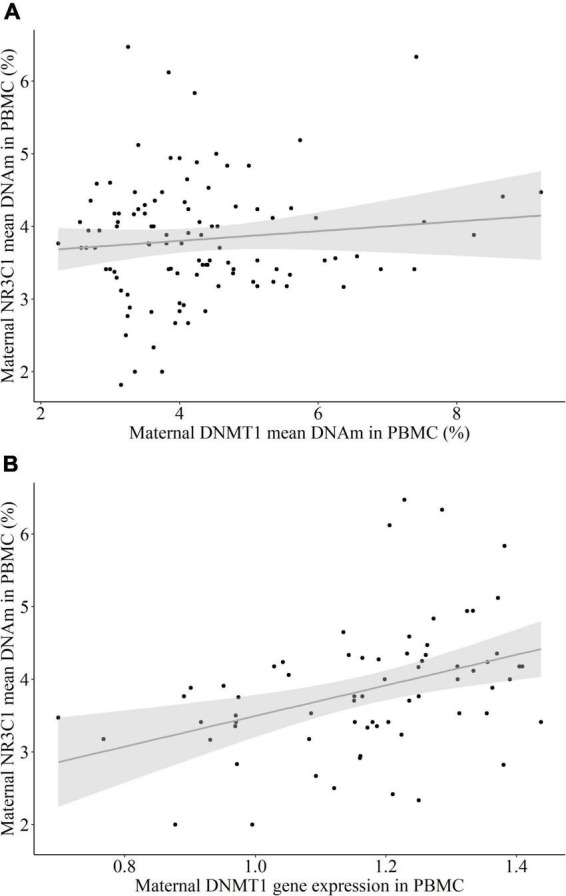
The association between NR3C1 mean DNA methylation (DNAm) and DNMT1 DNAm and relative gene expression in peripheral blood mononuclear cells (PBMC) of the mothers. **(A)** *DNMT1* mean DNAm did not have a significant correlation with *NR3C1* exon 1F mean DNAm (*r*_*s*_ = 0.031, *p* = 0.752). **(B)** *DNMT1* relative gene expression had a positive correlation with *NR3C1* exon 1F mean DNAm (*r*_*s*_ = 0.444, *p* < 0.001).

There was no significant correlation between *DNMT1* and *NR3C1* exon 1F mean DNAm (*r*_*s*_ = 0.031, *p* = 0.752, Figure 3A), but a trend toward significance between *DNMT1* mean DNAm and *NR3C1* relative gene expression (*r*_*s*_ = 0.215, *p* = 0.086). However, there was a significant positive correlation between relative *DNMT1* gene expression and *NR3C1* exon 1F mean DNAm (*r*_*s*_ = 0.444, *p* < 0.001, Figure 3B). No association was present between *DNMT1* and *NR3C1* relative gene expression levels (*r*_*s*_ = –0.189, *p* = 0.156).

#### Maternal DNA methyltransferase 1 methylation in buccal cells

**FIGURE 4:**
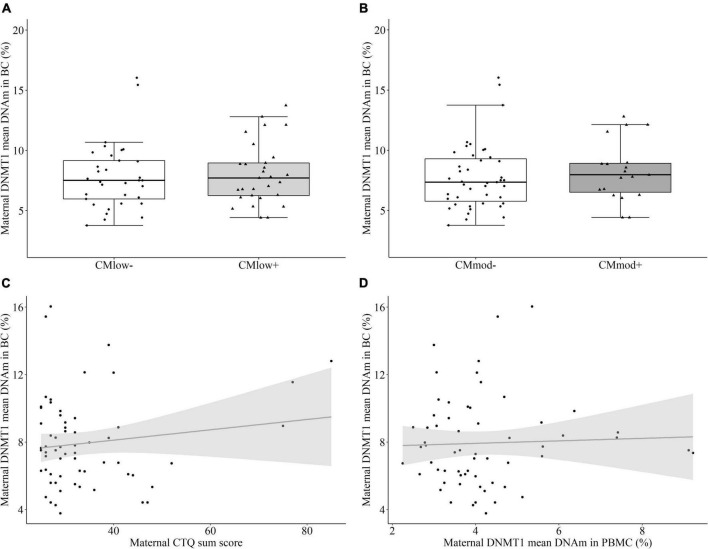
DNA methyltransferase 1 (DNMT1) mean DNA methylation (DNAm) in buccal cells (BC) of the mothers. **(A,B)** *DNMT1* mean DNA methylation did not differ in mothers categorized with their childhood maltreatment (CM) experiences depending on the low cut-off (CM_*low*–_ versus CM*_low+_*) or moderate cut-off (CM_*mod*–_ versus CM_*mod+*_). **(C)** *DNMT1* mean DNAm did not have a significant correlation with CTQ sum score (*r*_*s*_ = –0.086, *p* = 0.504). **(D)** There was no significant relationship between *DNMT1* mean DNAm in immune and buccal cells (*r*_*s*_ = –0.006, *p* = 0.965).

No significant difference in the mean percentage of buccal cell *DNMT1* DNAm between CM_*low*–_ (7.84 ± 2.78%) and CM_*low+*_ mothers (8.05 ± 2.54%, *T* = 0.328, *P* = 0.525 [0.375, 0.674], *p* = 0.744, Figure 4A), nor CM_*mod–*_ (7.80 ± 2.75%) and CM_*mod+*_ mothers (8.24 ± 2.47%, *T* = 0.829, *P* = 0.566 [0.405, 0.727], *p* = 0.412, Figure 4B) was found. There was no association between mean percentage buccal cell *DNMT1* DNAm and CTQ sum score (*r*_*s*_ = –0.086, *p* = 0.504, Figure 4C). These results did not change when covariates were introduced in robust regressions (see Section “Data processing and statistical analyses”). No relationship was present between the mean percentage *DNMT1* methylation levels of immune and buccal cells (*r*_*s*_ = –0.006, *p* = 0.965, Figure 4D). Buccal cell DNA (7.93 ± 2.65%) was significantly more methylated than PBMC DNA (4.34 ± 1.41%, W = 1853.0, *p* < 0.001). Analyses with single CpG units revealed no CM-status dependent difference in DNAm percentages after FDR correction (see Supplementary Tables 4, 5).

### Newborn methylation of DNA methyltransferase 1 according to maternal childhood maltreatment status and its relationship with maternal methylation

#### Newborn DNA methyltransferase 1 methylation in umbilical cord blood mononuclear cells

**FIGURE 5:**
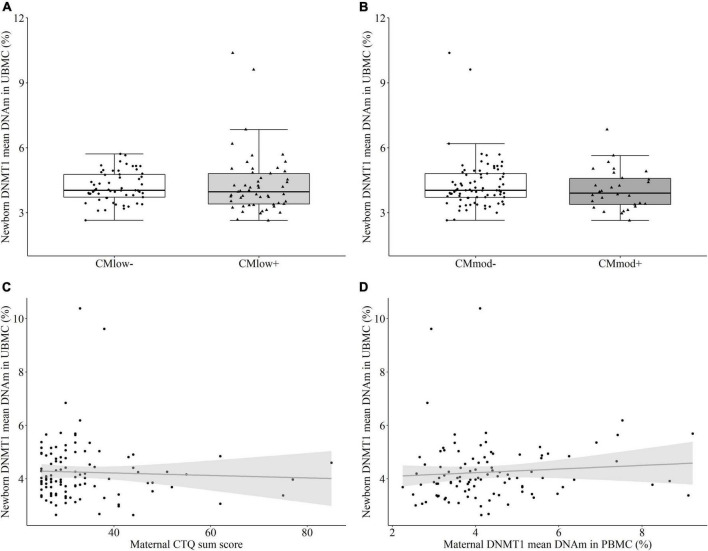
DNA methyltransferase 1 (DNMT1) mean DNA methylation (DNAm) in umbilical cord blood mononuclear cell (UBMC) of the newborns. **(A,B)** *DNMT1* mean DNA methylation did not differ in newborns with a maternal history of childhood maltreatment (CM) experiences depending on the low cut-off (CM_*low*–_ versus CM_*low+*_) or moderate cut-off (CM_*mod–*_ versus CM_*mod+*_). **(C)** *DNMT1* mean DNAm did not have a significant correlation with maternal CTQ sum score (*r*_*s*_ = –0.042, *p* = 0.662). **(D)** There was no significant correlation between *DNMT1* mean DNAm of mothers and newborns (*r*_*s*_ = 0.146, *p* = 0.143).

In newborns, maternal CM status did not predict differential mean percentage *DNMT1* methylation in UBMC according to neither the low CM cut-off [CM_*low*–_ (4.19 ± 0.71%) versus CM_*low+*_ (4.31 ± 1.45%), *T* = –0.801, *P* = 0.455 [0.343, 0.567], *p* = 0.425, Figure 5A] nor the moderate cut-off [CM_*mod*–_ (4.32 ± 1.18%) versus CM_*mod+*_ (4.07 ± 0.93%), *T* = –1.083, *P* = 0.430 [0.299, 0.560], *p* = 0.285, Figure 5B]. There was no association between newborn mean *DNMT1* methylation and maternal CTQ sum score (*r*_*s*_ = –0.042, *p* = 0.662, Figure 5C). The results did not change when tested for the effects of covariates (see Section “Data processing and statistical analyses”). There was no difference in female (4.40 ± 1.34%) and male (4.07 ± 0.76%) newborns in terms of mean *DNMT1* DNAm (*T* = –1.161, *P* = 0.436 [0.323, 0.544], *p* = 0.240). Single CpG units were not differentially methylated according to maternal CM status (Supplementary Tables 6, 7). Maternal and newborn mean *DNMT1* methylation also did not have a significant relationship (*r*_*s*_ = 0.146, *p* = 0.143, Figure 5D). However, DNAm percentage of nine single CpG units were correlated between mother-newborn dyads, independent of the CM status (Supplementary Table 8).

#### Newborn DNA methyltransferase 1 methylation in buccal cells

**FIGURE 6:**
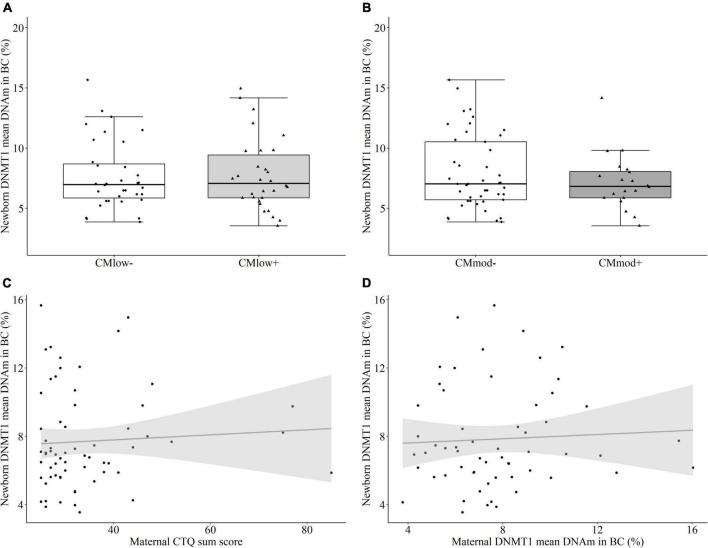
DNA methyltransferase 1 (DNMT1) mean DNA methylation (DNAm) in buccal cells (BC) of the newborns. **(A,B)** *DNMT1* mean DNA methylation did not differ in newborns with a maternal history of childhood maltreatment (CM) experiences depending on the low cut-off (CM_*low*–_ versus CM_*low+*_) or moderate cut-off (CM_*mod*–_ versus CM_*mod+*_). **(C)** *DNMT1* mean DNAm did not have a significant correlation with maternal CTQ sum score (*r*_*s*_ = 0.84, *p* = 0.505). **(D)** There was no significant correlation between *DNMT1* mean DNAm of mothers and newborns (*r*_*s*_ = 0.061, *p* = 0.647).

Similar to UBMC, there was no maternal CM-status dependent change in the mean percentage of *DNMT1* methylation in buccal cells according to neither the low CM cut-off [CM_*low*–_ (7.69 ± 2.84%) versus CM_*low+*_ (7.73 ± 2.98%), *T* = 0.052, *P* = 0.504 [0.356, 0.651], *p* = 0.959, Figure 6A] nor the moderate cut-off [CM_*mod*–_ (7.95 ± 3.09%) versus CM_*mod+*_ (7.16 ± 2.32%), *T* = –0.609, *P* = 0.454 [0.304, 0.605], *p* = 0.545, Figure 6B]. Maternal CTQ sum score was not associated with newborn buccal cell mean *DNMT1* methylation (*r*_*s*_ = 0.84, *p* = 0.505, Figure 6C). The results were similar when tested for the effects of covariates (see Section “Data processing and statistical analyses”). DNAm percentage of single CpG units did not differ according to the maternal CM status (Supplementary Tables 9, 10). In buccal cells, maternal and newborn mean *DNMT1* methylation (*r*_*s*_ = 0.061, *p* = 0.647, Figure 6D) as well as DNAm percentage of single CpG units (Supplementary Table 4) also did not have an association. Similar to mothers, no relationship was found between newborn buccal and immune cell mean *DNMT1* methylation (*r*_*s*_ = –0.058, *p* = 0.655), and buccal cell mean methylation (7.71 ± 2.88%) was higher than immune cell mean DNAm (4.25 ± 1.12%, W = 1868.0, *p* < 0.001).

## Discussion

This study aimed to explore the effects of different severities of maternal CM history on *DNMT1* methylation in immune and buccal cells of mother-newborn dyads. The results revealed associations between maternal *DNMT1* methylation and low as well as moderate CM in immune cells, such that mothers with a history of CM had significantly lower *DNMT1* mean DNAm than mothers without CM. CM load measured by CTQ sum score was associated with DNMT1 mean DNAm in a trend level (Figure 1C). CM status was also linked to the maternal *DNMT1* gene expression in PBMC, depending on the severity of CM: Mothers with a history of moderate CM (CM_*mod+*_) had higher relative *DNMT1* expression than mothers without a moderate CM history (CM_*mod*–_). However, no such difference was found between CM_*low*–_ and CM_*low+*_ mothers. CM history was not related to *DNMT1* DNAm in buccal cells. As for intergenerational analyses, maternal CM status was not found to be relevant in newborns’ *DNMT1* methylation levels neither in UBMC nor in buccal cells. There was also no association between maternal and infant mean *DNMT1* DNAm levels in immune or buccal cells. These results indicate no intergenerational transmission of low or moderate history of maternal CM in terms of *DNMT1* DNAm, both in terms of mean methylation and single CpG units.

### Maternal DNA methyltransferase 1 DNA methylation

The direction of the results for maternal PBMC supports most animal research and might explain some variance in the differential methylation profiles due to CM. Following diverse early life adversity paradigms, animal studies reported increased DNMT1 mRNA and protein levels in the brain (13–15). We have shown for the first time in humans, that CM is associated with decreased *DNMT1* DNAm in immune cells of women who recently gave birth. A history of moderate but not low CM was linked to higher relative *DNMT1* expression, compared to no or mild CM. Higher gene expression might mean higher enzymatic activity of DNMT1, meaning more methylation of hemimethylated strands as well as *de novo* CpG sites (12), depending on CM status. Adopting the traditional view of the relationship between DNA methylation and gene expression, the difference in the results according to CM severity might mean that the impact of CM on *DNMT1* DNAm is physiologically meaningful only for individuals with a moderate but not low CM status. However, apart from its influence on gene expression, DNAm might still impact the function of regulatory elements, other epigenetic mechanisms such as microRNA production and histone modification, as well as translational mechanisms such as isoform production (40–42). We have reported no association between mean *DNMT1* DNAm and relative *DNMT1* expression. Although DNAm of two CpG units did negatively correlate with relative expression (data not shown), new literature challenges the expected negative relationship between DNAm and gene expression, reporting null and even positive associations (43, 44). Additionally, the difference in mean *DNMT1* DNAm between groups with and without CM is very low, with a difference of 0.4% in low CM and 0.6% in moderate CM groups. The physiological relevance of this small difference might be challenged and should be treated with caution (45). Nevertheless, CpG sites around the promoter region are known to be rarely methylated (41), therefore a big difference in methylation depending on CM should not be expected in this highly conserved gene.

In complex phenotypes (i.e., psychopathology) that might be influenced by certain environmental factors such as early life adversity, DNAm was proposed to create subtle differences between individuals with and without a certain environmental exposure which might explain some of the variance of the phenotype (42). Sipahi et al. reported 0.8% difference in DNMT1 DNAm in PTSD cases before and after trauma (17), which is close to our findings in a cohort with mostly low CM experiences. Their reported mean DNAm values were around 3–4%, which were also close to our results. They reported a change opposite to the direction of our results, with an increased DNMT1 DNAm after trauma. This contradiction might be due to the time course of the trauma, adulthood versus childhood, which might be associated with differing pathologies (46), thus physiological correlates of these two might differ.

Apart from mean DNAm, the effects of single CpG unit methylation, which differ up to 7% in a unit that is located in a predicted TSS between groups with different CM_*mod*_ status (Supplementary Tables 2, 3), might also be important since they can influence gene regulation and transcription factor binding. CpG units that seemed to be methylated less in CM+ groups are mostly in regulatory regions in both low and moderate CM categorizations. Considering the decreasing DNAm levels with increased CM severity, our results seem to be a sign of allostatic load for mothers with a history low and moderate CM, and they might also be physiologically relevant at least for mothers with moderate CM, and explain some findings in the literature regarding the CM-dependent differential DNAm. In line with this interpretation, psychopathological conditions with allostatic overload (47) have been previously associated with DNMT1 overactivation (19), and can be treated with antidepressants that have reported to reduce DNMT1 activity (21, 22).

We have reported a positive association between *DNMT1* relative gene expression and *NR3C1* exon 1F mean DNAm. It hints toward a link between mechanisms of stress-response regulation and DNA methylation, as previously suggested (8–10). As *NR3C1* exon 1F DNAm has been repeatedly linked with early life adversity (5), it is possible that with increased levels of stress and allostatic load, glucocorticoid receptor–glucocorticoid complexes impact transcription of *DNMT1*, which in turn contributes to *de novo* methylation of the glucocorticoid receptor gene. Nevertheless, the meaning of this association and the direction of a causal relationship is not exactly clear and cannot be properly interpreted using our cross-sectional design. The relationship between the regulators of DNAm machinery and the stress-response systems should be investigated in longitudinal designs, including the remaining elements of these processes.

### Intergenerational aspects of childhood maltreatment-related DNA methyltransferase 1 DNA methylation

In line with our previous results (7) concerning stress-response genes, we did not find any evidence concerning an intergenerational transmission of maternal CM-associated DNAm changes. There was no differential methylation due to the maternal history of CM in *DNMT1* DNAm in newborn UBMC of neither single CpG units nor mean percentage DNAm of these units. Our sample consisted of mostly healthy women who recently gave birth and their newborns, which enabled us to eliminate the effects of important confounders such as familial environment and parenting behavior that can be also influenced by parental history of CM (27). Our results suggest no intergenerational transmission, at least not as a result of incomplete erosion of CM-related changes in epigenetic signatures in parental gametes. Our results also do not suggest a maternal CM-related influence in fetus development or gestational biology in *DNMT1* DNAm. Results might differ in a cohort with a maternal history of severe CM, and high prenatal stress associated with CM. Furthermore, maternal and newborn mean *DNMT1* DNAm in immune cells were not associated, but there were associations between methylation levels in newborns and mothers at a single CpG unit level independently of CM status (Supplementary Table 8). Therefore, there might be transmission, or rather inheritance, of some methylation markers independent of any maternal history of childhood trauma. This “transmission” process might be explained by regular physiological processes, such as these regions being protected from demethylation during zygote formation, or being reestablished during fetal development (48). Lastly, different results might be obtained with newborn PBMC compared to UBMC, due to differences in cell type and composition in these cell populations (49).

### Tissue specificity of childhood maltreatment-related DNA methyltransferase 1 DNA methylation

Our results show the importance of tissue selection in DNAm studies. In contrast to our results with maternal PBMC, we did not find any CM related changes in *DNMT1* DNAm in either single CpG units or mean percentage in the buccal cells of mothers. The levels of the methylation signatures in immune versus buccal cells were not correlated either. Studying buccal cells have its advantages compared to immune cells, such as they are more accessible, less invasive, and cheaper to collect. Although they also have a few subtypes (50), buccal cells might be more uniform in terms of cell composition which can give them a more homogeneous DNAm signature, compared to PBMCs which include several subtypes of lymphocytes, monocytes and natural killer cells. However, epigenetic modifications can be tissue and cell type-specific, and one should be mindful about studying the impact of a certain trait or environmental factor on DNAm in a certain tissue. In this study, we hypothesized that CM-related physiological changes such as alterations in immune and neuroendocrine systems (3, 51) might lead to alterations in DNAm of an important regulator of DNAm, DNMT1. These alterations should be relevant to immune cells but not necessarily to buccal cells. To our knowledge, no study so far investigated cross-tissue comparisons of DNAm profiles of stress-related genes or *DNMT1* due to a psychological stressor, but one genome-wide DNAm study reported an association between buccal cell and blood DNAm levels of one CpG site linked to CM-related cortisol stress reactivity (52), and another study reported neural correlates of *NR3C1* buccal cell methylation (53). Future studies should focus on cross-tissue associations and factors related to differential methylation in tissues.

### Limitations

This study has several limitations. Firstly, although immune cells might be more relevant targets compared to buccal cells, epigenetic changes might be cell type-specific, therefore changing cell compositions between individuals might lead to questionable results since it is not clear whether the results are due to epigenetic modifications or cell-type-specific proliferation. Our models including lymphocyte to monocyte ratios did not change our main results but we could not control for subtypes of these cells as the data was not available. Future studies should investigate how different immune cell subpopulations may impact difference in DNAm levels. One factor that can lead to changes in cell composition is certainly the postpartum state of the sample, which is known to have unique immunological alterations due to parturition (54) that might also compromise the generalizability of our results. However, this period is the best to study intergenerational transmission effects in humans, thereby it was chosen. Days since parturition was used in the robust regression models as a predictor in order to control for changing immune system within the postpartum period. Breastfeeding status, another postpartum variable that can influence immune system and overall metabolism (55, 56) thus our results, could not be assessed in this cohort. We also cannot generalize these results to a male cohort or an older cohort. Additionally, a retrospective self-report measure (CTQ) was used to assess CM. These measures were shown to be only in modest agreement with prospective measures, and report a smaller number of traumatic events compared to prospective measures (57), therefore results of our study might differ with a prospective measure of CM. This sample was limited in terms of CM severity, with mothers with CM having experienced mostly low CM. Nevertheless, even in this healthy population with low CM, we have observed CM-related *DNMT1* DNAm changes in immune cells.

## Conclusion

We conclude that CM-associated alterations in *DNMT1* DNAm do exist and might have physiological relevance, especially in individuals with a more severe CM load. Increased DNMT1 expression due to demethylation linked to CM experiences with at least moderate severity might explain differential methylation profiles due to CM to some extent, especially in stress response genes as shown in *NR3C1* DNAm. Our results might demonstrate the effect of stress-response elements on DNMT1 expression thus might reflect allostatic load in individuals with CM. The direction of the PBMC results supports most animal research about DNMT1 levels following adversity, and the pathophysiological conditions that have been characterized with allostatic overload. Furthermore, the CM-related differential methylation in single CpG units might impact the function of regulatory elements and translational mechanisms. Our lack of significant findings in buccal cells shows the tissue-specific effects of CM on *DNMT1* DNAm. Importantly, no evidence for intergenerational transmission of *DNMT1* DNAm was found, which complements previous findings reporting against transmission of DNAm signatures in cohorts with similar maternal CM experiences.

## Data availability statement

The original contributions presented in this study are included in the article/Supplementary material, further inquiries can be directed to the corresponding author.

## Ethics statement

The studies involving human participants were reviewed and approved by Ulm University. Written informed consent to participate in this study was provided by the participants themselves or the participants’ legal guardians.

## Author contributions

AK and ITK conceptualized the study. ITK acquired the funding and organized and supervised the data collection with her team. LRF and AK designed the methylation targets. LRF, AMG, and RNM contributed to biological analyses. RNM performed the statistical analyses, interpreted the findings, and wrote the first draft of the manuscript. All authors provided critical input to the manuscript and revised and approved the final version.
