# Supplementary material for: A history of childhood maltreatment is associated with altered DNA methylation levels of DNA methyltransferase 1 in maternal but not neonatal mononuclear immune cells
Source: Front Psychiatry. 2022 Nov 10;13:945343. doi: 10.3389/fpsyt.2022.945343 (PMC9685310; doi:10.3389/fpsyt.2022.945343)
Supplement: Supplementary file 1 [file Data_Sheet_1.PDF]

Supplementary Information: A history of childhood maltreatment is associated with altered methylation levels of DNMT1 in maternal but not in neonatal mononuclear immune cells

R. Nehir Mavioglu[1*], Laura Ramo-Fernandez[1], Anja M. Gumpp[1], Iris-Tatjana Kolassa[1], Alexander Karabatsiakis[1,2]

[1]Department of Clinical & Biological Psychology, Institute of Psychology and Education, Ulm University, Ulm, Germany

[2]Department of Clinical Psychology, Institute of Psychology, University of Innsbruck, Innsbruck, Austria

*** Correspondence:** R. Nehir Mavioglu, nehir.mavioglu@uni-ulm.de

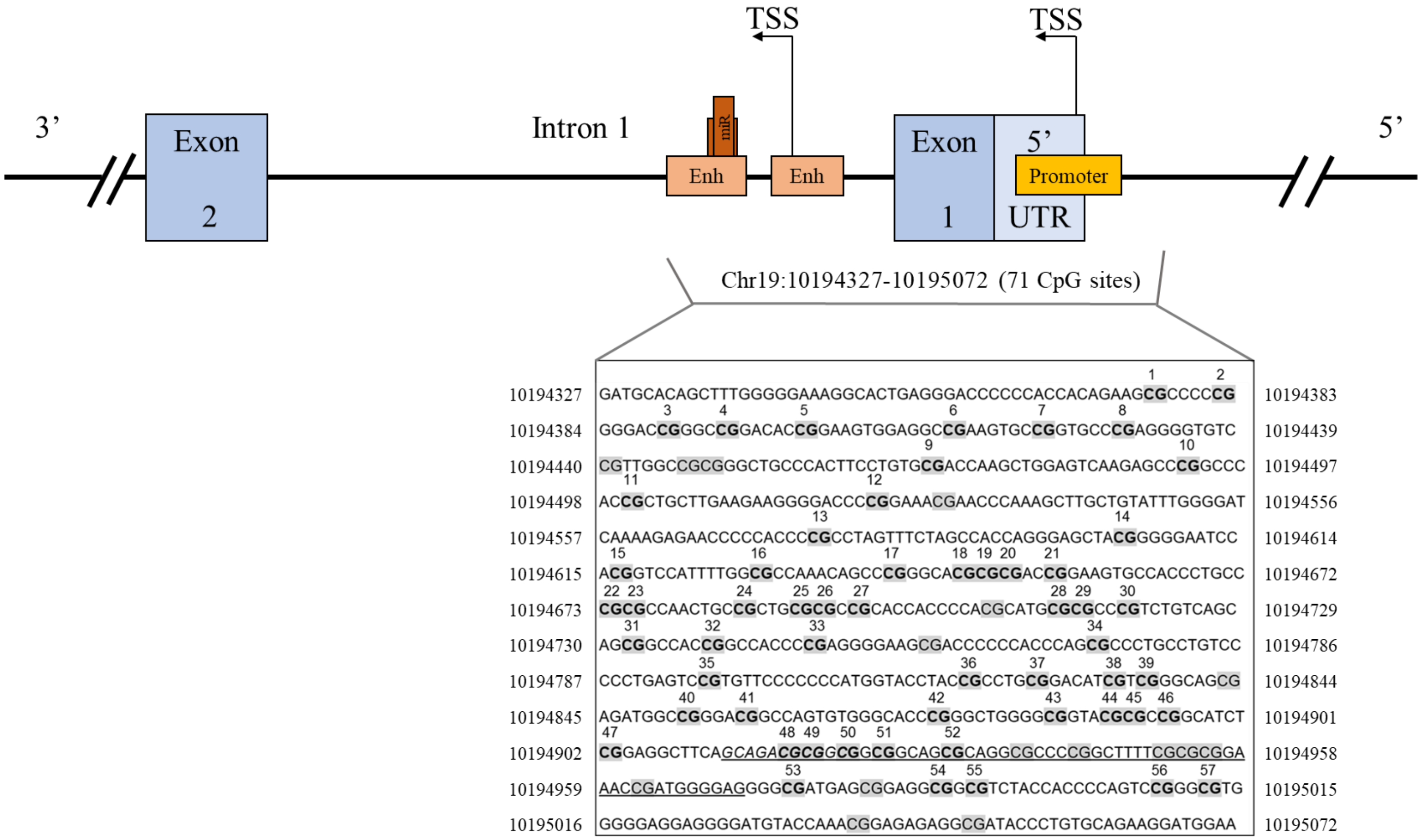

Supplementary Figure 1: Depiction of *DNMT1* gene in schematic form, and the selected sequence (GRCh38/hg38) for DNA methylation analyses. Since the *DNMT1* gene is on the negative strand, the schema is shown from 3’ to 5’. Genomic regions such as 5’ untranslated region (5’UTR), exons 1 and 2, and intron 1 are depicted. For exon 1, 5’UTR and the coding sequence of the exon (depicted “Exon 1”) were drawn separately. Predicted regulatory regions such as promoter (Eukaryotic promoter database (EPD; 1), transcription start site (TSS; refTSS (2)), enhancers (Enh; ENCODE (3)) and microRNA targets (miR; TarBase (4)) were included in the schema. Start and end locations for each row of the sequence were added on the sides of the sequence box. In the sequence box, the predicted promoter region (EPD (1)) is underlined, and the predicted TSS (EPD (1)) is italicized. All CpG sites are highlighted in grey. CpG sites marked in bold and numbered are successfully detected in methylation analyses.

Supplementary Table 1: Function and location of the genomic regions in the sequence used, and the corresponding CpG units

**Function of the genomic region (database, accession ID)**	**Genomic location (Chr 19, GrCh38/hg38)**	**Corresponding CpG units**
Enhancer<br>(ENCODE (3), EH38E1938037)	10194367 - 10194586	DNMT1.1.2, DNMT1.3.4, DNMT1.5, DNMT1.6, DNMT1.7.8, DNMT1.9, DNMT1.10, DNMT1.11, DNMT1.12, DNMT1.13
MicroRNA target<br>(TarBase (4), MIMAT0019219)	10194515 - 10194543	DNMT1.12
MicroRNA target<br>(TarBase (4), MIMAT0002875)	10194517 - 10194535	DNMT1.12
Transcription start site<br>(refTSS (2), hg_179088.1)	10194629 - 10194666	DNMT1.16, DNMT1.17, DNMT1.18.19.20, DNMT1.21.52
Enhancer<br>(ENCODE (3), EH38E1938038)	10194630 - 10194786	DNMT1.16, DNMT1.17, DNMT1.18.19.20, DNMT1.21.52, DNMT1.22.23, DNMT1.24.25.26.27, DNMT1.28.29.30, DNMT1.31, DNMT1.15.32.47, DNMT1.33, DNMT1.34
Exon - coding sequence<br>(NCBI (5), gene ID 1786)	10194820 - 10194899	DNMT1.36.37, DNMT1.38.39, DNMT1.40, DNMT1.41, DNMT1.42.43, DNMT1.44.45.46
5'UTR<br>(NCBI (5), gene ID 1786)	10194900 - 10194954	DNMT1.15.32.47, DNMT1.48.49.50.51, DNMT1.21.52
Transcription start site<br>(refTSS (2), hg_179089.1)	10194906 - 10194970	DNMT1.48.49.50.51, DNMT1.21.52
Promoter<br>(EPD (1), DNMT1_1)	10194913 - 10194972	DNMT1.48.49.50.51, DNMT1.21.52

Supplementary Table 2: Descriptives and comparisons of individual CpG unit methylation percentages in immune cells of mothers by history of childhood maltreatment, low cut-off

**CpG unit**	**N**	**CM$_{low-}$**		**CM$_{low+}$**		**Estimator (LCI - UCI)**	**SE**	**T**	***p*$_{adj}$**
		**Mean**	***SD***	**Mean**	***SD***				
DNMT1.1.2	109	2.44	3.82	2.56	3.31	0.5614 (0.4577-0.6651)	0.052	1.174	0.370
DNMT1.3.4	109	.56	.89	.98	2.69	0.4754 (0.3799-0.5709)	0.048	-0.511	0.697
**DNMT1.5**	**93**	**6.35**	**5.13**	**3.70**	**3.71**	**0.339 (0.2275-0.4506)**	**0.056**	**-2.867**	**0.017**
**DNMT1.6**	**107**	**2.54**	**2.43**	**1.64**	**2.54**	**0.3313 (0.2286-0.434)**	**0.052**	**-3.258**	**0.007**
DNMT1.7.8	108	2.23	.85	2.48	1.64	0.5155 (0.4142-0.6167)	0.051	0.303	0.799
DNMT1.9	100	3.13	1.62	3.12	1.82	0.4836 (0.3704-0.5968)	0.057	-0.288	0.799
**DNMT1.10**	**106**	**3.96**	**1.42**	**3.11**	**2.01**	**0.3364 (0.2326-0.4401)**	**0.052**	**-3.131**	**0.009**
DNMT1.11	108	1.87	1.19	1.89	3.49	0.4009 (0.2961-0.5057)	0.053	-1.874	0.157
DNMT1.12	107	1.16	1.19	1.04	1.16	0.4723 (0.3654-0.5793)	0.054	-0.513	0.697
**DNMT1.13**	**101**	**6.71**	**5.72**	**3.83**	**3.81**	**0.2498 (0.1504-0.3492)**	**0.050**	**-5.009**	**< .001**
DNMT1.14.53	106	1.27	2.15	1.76	2.33	0.5646 (0.4615-0.6678)	0.052	1.243	0.365
**DNMT1.15.32.47**	**107**	**5.16**	**2.31**	**4.05**	**2.60**	**0.3227 (0.2189-0.4264)**	**0.052**	**-3.389**	**0.005**
DNMT1.16	107	2.55	2.19	2.16	2.07	0.4335 (0.3232-0.5437)	0.056	-1.198	0.370
DNMT1.17	109	.19	.77	.53	1.26	0.5516 (0.4852-0.6181)	0.034	1.541	0.270
DNMT1.18.19.20	109	31.67	12.20	28.35	13.05	0.4556 (0.3449-0.5663)	0.056	-0.795	0.571
**DNMT1.21.52**	**103**	**9.04**	**8.10**	**6.93**	**14.18**	**0.2741 (0.1698-0.3785)**	**0.053**	**-4.305**	**< .001**
**DNMT1.22.23**	**106**	**5.35**	**3.94**	**9.51**	**9.08**	**0.7003 (0.5975-0.8032)**	**0.052**	**3.863**	**0.002**
**DNMT1.24.25.26.27**	**105**	**2.88**	**1.75**	**4.48**	**4.76**	**0.6516 (0.5465-0.7567)**	**0.053**	**2.861**	**0.017**
DNMT1.28.29.30	99	3.31	1.42	3.25	2.39	0.4363 (0.3226-0.55)	0.057	-1.114	0.391
DNMT1.31	106	.25	.81	.61	2.10	0.5424 (0.4751-0.6096)	0.034	1.250	0.365
DNMT1.33	104	3.56	4.01	2.77	3.63	0.4076 (0.2972-0.5179)	0.056	-1.664	0.227
DNMT1.34	92	1.83	2.00	2.25	2.14	0.5636 (0.4457-0.6815)	0.059	1.073	0.398
DNMT1.35	101	2.49	2.25	2.33	2.31	0.4704 (0.3572-0.5837)	0.057	-0.518	0.697
DNMT1.36.37	106	4.62	3.64	4.05	2.28	0.5045 (0.392-0.617)	0.057	0.079	0.937
**DNMT1.38.39**	**107**	**6.25**	**5.69**	**3.43**	**2.35**	**0.3032 (0.2047-0.4017)**	**0.050**	**-3.963**	**0.001**
**DNMT1.40**	**109**	**.85**	**1.74**	**1.86**	**2.61**	**0.6253 (0.5277-0.723)**	**0.049**	**2.545**	**0.033**
**DNMT1.41**	**109**	**7.77**	**7.56**	**9.18**	**19.29**	**0.3617 (0.2564-0.4669)**	**0.053**	**-2.608**	**0.031**
DNMT1.42.43	106	2.33	2.76	2.18	1.78	0.5328 (0.4217-0.6439)	0.056	0.585	0.697
**DNMT1.44.45.46**	**109**	**14.65**	**8.87**	**9.79**	**5.82**	**0.309 (0.208-0.4101)**	**0.051**	**-3.747**	**0.002**
DNMT1.48.49.50.51	108	1.55	3.36	1.77	3.79	0.5217 (0.4176-0.6257)	0.053	0.413	0.751
DNMT1.54.55	103	3.36	2.88	3.96	3.51	0.5777 (0.4651-0.6904)	0.057	1.370	0.338
DNMT1.56.57	108	1.17	2.02	1.55	2.46	0.5713 (0.4667-0.6758)	0.053	1.352	0.338

Group comparisons are conducted with Brunner-Munzel tests (see 2.6 for details). Multiple testing correction by false discovery rate was conducted and only adjusted *p* values (p_{adj}) were reported. Tests with $p_{adj} < .05$ were marked as bold. CM: childhood maltreatment, SD: standard deviation, LCI: lower 95% confidence interval, UCI: Upper 95% confidence interval, SE: standard error, T: Brunner-Munzel test statistic

Supplementary Table 3: Descriptives and comparisons of individual CpG unit methylation percentages in immune cells of mothers by history of childhood maltreatment, moderate cut-off

CpG unit	N	CM_{mod-} Mean	CM_{mod-} *SD*	CM_{mod+} Mean	CM_{mod+} *SD*	Estimator (LCI - UCI)	SE	T	p_{adj}
DNMT1.1.2	109	2.37	3.27	2.82	4.16	0.5439 (0.4274-0.6604)	0.058	0.754	0.765
DNMT1.3.4	109	0.53	0.82	1.36	3.47	0.4773 (0.3659-0.5886)	0.055	-0.410	0.913
DNMT1.5	93	5.35	4.97	4.19	3.65	0.4461 (0.3185-0.5738)	0.064	-0.845	0.714
DNMT1.6	107	2.23	2.62	1.72	2.29	0.4152 (0.2972-0.5332)	0.059	-1.437	0.356
DNMT1.7.8	108	2.39	1.40	2.28	1.14	0.5016 (0.3834-0.6199)	0.059	0.028	0.992
DNMT1.9	100	3.04	1.55	3.29	2.07	0.5208 (0.3948-0.6468)	0.063	0.331	0.913
DNMT1.10	106	3.79	1.81	2.87	1.57	0.3417 (0.2212-0.4622)	0.060	-2.641	0.071
DNMT1.11	108	1.77	1.17	2.12	4.46	0.3992 (0.2804-0.518)	0.059	-1.701	0.246
DNMT1.12	107	1.15	1.25	0.97	0.97	0.4798 (0.3664-0.5932)	0.057	-0.356	0.913
**DNMT1.13**	**101**	**5.79**	**5.23**	**3.97**	**4.35**	**0.3104 (0.1884-0.4324)**	**0.061**	**-3.124**	**0.048**
DNMT1.14.53	106	1.61	2.37	1.30	1.93	0.4708 (0.3567-0.585)	0.057	-0.512	0.913
DNMT1.15.32.47	107	4.84	2.42	4.00	2.67	0.3638 (0.246-0.4817)	0.059	-2.313	0.097
DNMT1.16	107	2.57	2.20	1.81	1.86	0.3856 (0.2633-0.508)	0.061	-1.877	0.193
DNMT1.17	109	0.24	0.85	0.67	1.41	0.5712 (0.4859-0.6565)	0.042	1.681	0.246
DNMT1.18.19.20	109	30.79	12.68	27.97	12.72	0.4623 (0.3458-0.5788)	0.059	-0.645	0.834
**DNMT1.21.52**	**103**	**10.23**	**12.43**	**2.94**	**8.57**	**0.2318 (0.1361-0.3275)**	**0.048**	**-5.594**	**< .001**
DNMT1.22.23	106	6.21	4.36	10.64	11.18	0.649 (0.531-0.7671)	0.059	2.527	0.076
DNMT1.24.25.26.27	105	3.71	4.19	3.78	2.51	0.5593 (0.4319-0.6867)	0.064	0.934	0.668
DNMT1.28.29.30	99	3.26	1.82	3.33	2.31	0.4836 (0.349-0.6181)	0.067	-0.246	0.929
DNMT1.31	106	0.34	0.95	0.67	2.63	0.4945 (0.4187-0.5704)	0.038	-0.145	0.977
DNMT1.33	104	3.38	4.00	2.61	3.36	0.4445 (0.3264-0.5626)	0.059	-0.939	0.668
DNMT1.34	92	2.08	2.21	2.03	1.81	0.5239 (0.3948-0.6531)	0.065	0.371	0.913
DNMT1.35	101	2.55	2.32	2.07	2.16	0.4322 (0.3063-0.558)	0.063	-1.082	0.607
DNMT1.36.37	106	4.49	3.32	3.94	2.12	0.4994 (0.3777-0.6211)	0.061	-0.010	0.992
DNMT1.38.39	107	5.37	5.03	3.38	2.34	0.3548 (0.2463-0.4633)	0.055	-2.666	0.071
DNMT1.40	109	1.03	1.77	2.18	3.06	0.6128 (0.4978-0.7279)	0.057	1.968	0.185
DNMT1.41	109	10.18	16.68	4.64	8.34	0.3503 (0.2375-0.463)	0.056	-2.660	0.071
DNMT1.42.43	106	2.34	2.45	2.03	1.86	0.4782 (0.3539-0.6025)	0.062	-0.352	0.913
DNMT1.44.45.46	109	13.03	8.56	10.00	5.14	0.3686 (0.261-0.4762)	0.054	-2.425	0.079
DNMT1.48.49.50.51	108	1.71	3.46	1.58	3.89	0.5026 (0.3934-0.6119)	0.055	0.048	0.992
DNMT1.54.55	103	3.36	2.81	4.43	4.00	0.6123 (0.4962-0.7284)	0.058	1.932	0.185
DNMT1.56.57	108	0.01	0.02	0.01	0.03	0.4865 (0.3725-0.6004)	0.057	-0.237	0.929

Group comparisons are conducted with Brunner-Munzel tests (see 2.6 for details). Multiple testing correction by false discovery rate was conducted and only adjusted *p* values (p_{adj}) were reported. Tests with $p_{adj} < .05$ were marked as bold. CM: childhood maltreatment, SD: standard deviation, LCI: lower 95% confidence interval, UCI: Upper 95% confidence interval, SE: standard error, T: Brunner-Munzel test statistic

Supplementary Table 4: Descriptives and comparisons of individual CpG unit methylation percentages in buccal cells of mothers by history of childhood maltreatment, low cut-off

CpG unit	N	CM_{low-}		CM_{low+}		Estimator (LCI - UCI)	SE	T	p_{adj}
		Mean	*SD*	Mean	*SD*				
DNMT1.1.2	69	1.57	0.88	1.50	0.51	0.5071 (0.3815-0.6328)	0.063	0.114	0.960
DNMT1.3.4	69	0.94	0.59	0.79	0.48	0.4471 (0.3406-0.5536)	0.053	-0.992	0.893
DNMT1.5	52	5.65	4.44	4.65	3.43	0.443 (0.2807-0.6054)	0.081	-0.705	0.933
DNMT1.6	67	1.26	2.19	1.03	1.12	0.5147 (0.3798-0.6497)	0.068	0.218	0.960
DNMT1.7.8	69	3.23	0.88	3.06	0.78	0.4534 (0.33-0.5767)	0.062	-0.755	0.933
DNMT1.9	69	3.29	1.71	2.53	1.16	0.3559 (0.2279-0.4839)	0.064	-2.249	0.434
DNMT1.10	69	5.63	1.11	6.06	1.10	0.5836 (0.4515-0.7157)	0.066	1.265	0.653
DNMT1.11	69	2.63	0.73	2.65	0.92	0.4941 (0.3634-0.6248)	0.066	-0.090	0.960
DNMT1.12	68	3.69	2.49	4.18	2.48	0.5636 (0.4253-0.7019)	0.069	0.920	0.893
DNMT1.13	62	7.76	5.22	6.83	4.92	0.4556 (0.3065-0.6047)	0.075	-0.596	0.933
DNMT1.14.53	61	1.06	1.79	1.76	2.72	0.5603 (0.4253-0.6954)	0.067	0.895	0.893
DNMT1.15.32.47	62	5.67	4.52	5.41	3.52	0.4822 (0.3311-0.6334)	0.076	-0.235	0.960
DNMT1.16	61	1.30	1.24	1.54	1.99	0.4968 (0.3507-0.6428)	0.073	-0.045	0.965
DNMT1.17	62	0.00	0.00	0.21	0.77	0.5345 (0.4854-0.5835)	0.024	1.440	0.653
DNMT1.18.19.20	61	40.25	13.16	44.66	11.38	0.6185 (0.4735-0.7636)	0.073	1.636	0.653
DNMT1.21.52	52	30.61	36.91	35.58	42.78	0.5268 (0.3598-0.6938)	0.083	0.324	0.960
DNMT1.22.23	59	6.19	2.71	8.00	3.91	0.6227 (0.4753-0.7701)	0.074	1.670	0.653
DNMT1.24.25.26.27	57	4.33	2.07	5.19	2.56	0.5994 (0.4472-0.7516)	0.076	1.310	0.653
DNMT1.28.29.30	61	4.72	2.10	4.24	1.24	0.4677 (0.3215-0.6139)	0.073	-0.443	0.960
DNMT1.31	61	0.16	0.63	0.07	0.37	0.4855 (0.4299-0.541)	0.028	-0.525	0.933
DNMT1.34	57	2.74	2.38	2.92	2.46	0.5298 (0.3728-0.6867)	0.078	0.381	0.960
DNMT1.35	57	4.58	4.29	4.92	5.11	0.5118 (0.3522-0.6713)	0.079	0.148	0.960
DNMT1.36.37	62	3.06	2.56	3.21	3.12	0.4896 (0.3391-0.64)	0.075	-0.139	0.960
DNMT1.38.39	62	9.39	9.59	11.14	8.13	0.6233 (0.4796-0.767)	0.072	1.717	0.653
DNMT1.40	62	2.00	3.91	1.97	4.26	0.5366 (0.3981-0.675)	0.069	0.528	0.933
DNMT1.41	62	57.33	36.53	51.97	31.30	0.4431 (0.2942-0.5919)	0.074	-0.766	0.933
DNMT1.42.43	62	6.61	5.24	7.41	6.65	0.5413 (0.3911-0.6915)	0.075	0.550	0.933
DNMT1.44.45.46	62	18.30	11.83	20.76	10.13	0.605 (0.4598-0.7503)	0.073	1.446	0.653
DNMT1.48.49.50.51	59	2.31	3.95	1.56	1.69	0.4809 (0.3344-0.6274)	0.073	-0.261	0.960
DNMT1.54.55	62	4.45	2.93	3.69	2.98	0.4018 (0.2556-0.548)	0.073	-1.346	0.653
DNMT1.56.57	62	1.27	1.33	0.48	0.91	0.3119 (0.1878-0.436)	0.062	-3.033	0.112

Group comparisons are conducted with Brunner-Munzel tests (see 2.6 for details). Multiple testing correction by false discovery rate was conducted and only adjusted *p* values (p_{adj}) were reported. Tests with $p_{adj} < .05$ were marked as bold. CM: childhood maltreatment, SD: standard deviation, LCI: lower 95% confidence interval, UCI: Upper 95% confidence interval, SE: standard error, T: Brunner-Munzel test statistic

Supplementary Table 5: Descriptives and comparisons of individual CpG unit methylation percentages in buccal cells of mothers by history of childhood maltreatment, moderate cut-off

CpG unit	N	CM_{mod-} Mean	CM_{mod-} *SD*	CM_{mod+} Mean	CM_{mod+} *SD*	Estimator (LCI - UCI)	SE	T	p_{adj}
DNMT1.1.2	69	1.57	0.81	1.48	0.51	0.4901 (0.3615-0.6186)	0.064	-0.155	0.938
DNMT1.3.4	69	0.91	0.55	0.78	0.52	0.4499 (0.3317-0.5682)	0.059	-0.856	0.880
DNMT1.5	52	5.76	3.99	4.00	3.73	0.3685 (0.1952-0.5417)	0.085	-1.548	0.510
DNMT1.6	67	1.09	2.03	1.27	0.98	0.6424 (0.5107-0.7741)	0.066	2.164	0.184
DNMT1.7.8	69	3.11	0.88	3.22	0.74	0.5369 (0.4037-0.67)	0.066	0.558	0.938
DNMT1.9	69	3.07	1.62	2.61	1.20	0.4069 (0.2706-0.5432)	0.068	-1.370	0.547
DNMT1.10	69	5.72	1.09	6.09	1.16	0.5666 (0.4317-0.7016)	0.067	0.989	0.780
DNMT1.11	69	2.59	0.80	2.74	0.86	0.5132 (0.3695-0.657)	0.071	0.186	0.938
DNMT1.12	68	3.96	2.84	3.86	1.52	0.5198 (0.3799-0.6596)	0.070	0.283	0.938
DNMT1.13	62	7.84	4.79	6.16	5.59	0.388 (0.2151-0.5609)	0.084	-1.331	0.549
DNMT1.14.53	61	1.14	1.83	1.95	3.06	0.5482 (0.3919-0.7046)	0.077	0.631	0.938
DNMT1.15.32.47	62	5.30	4.13	6.11	3.93	0.5624 (0.3949-0.73)	0.082	0.759	0.937
DNMT1.16	61	1.26	1.18	1.78	2.37	0.5194 (0.3422-0.6965)	0.086	0.225	0.938
DNMT1.17	62	0.14	0.64	0.00	0.00	0.4767 (0.444-0.5095)	0.016	-1.431	0.547
DNMT1.18.19.20	61	40.02	12.19	47.47	11.73	0.693 (0.551-0.835)	0.071	2.739	0.136
DNMT1.21.52	52	32.68	38.74	33.47	42.41	0.4351 (0.2246-0.6457)	0.100	-0.646	0.938
DNMT1.22.23	59	6.43	3.12	8.47	3.73	0.6723 (0.5124-0.8321)	0.078	2.204	0.184
DNMT1.24.25.26.27	57	4.28	2.21	5.72	2.35	0.6802 (0.5261-0.8343)	0.076	2.376	0.184
DNMT1.28.29.30	61	4.55	2.03	4.37	0.90	0.532 (0.3863-0.6776)	0.073	0.440	0.938
DNMT1.31	61	0.12	0.55	0.11	0.46	0.5019 (0.4392-0.5645)	0.031	0.061	0.952
DNMT1.34	57	2.73	2.23	3.06	2.86	0.5198 (0.3256-0.7141)	0.093	0.212	0.938
DNMT1.35	57	4.55	3.91	5.18	6.15	0.4625 (0.2656-0.6594)	0.095	-0.395	0.938
DNMT1.36.37	62	3.07	2.48	3.26	3.53	0.47 (0.2902-0.6498)	0.087	-0.343	0.938
DNMT1.38.39	62	9.09	8.66	12.74	9.18	0.6671 (0.5196-0.8145)	0.073	2.289	0.184
DNMT1.40	62	2.09	3.60	1.74	5.01	0.4229 (0.2777-0.5681)	0.072	-1.075	0.747
DNMT1.41	62	55.88	35.13	52.42	32.13	0.4639 (0.3078-0.62)	0.077	-0.466	0.938
DNMT1.42.43	62	6.72	4.93	7.58	7.80	0.5061 (0.3351-0.6771)	0.084	0.073	0.952
DNMT1.44.45.46	62	18.02	10.91	22.68	10.95	0.6438 (0.4908-0.7969)	0.076	1.903	0.287
DNMT1.48.49.50.51	59	2.02	3.53	1.83	1.98	0.5325 (0.3688-0.6962)	0.080	0.405	0.938
DNMT1.54.55	62	4.16	2.85	3.95	3.24	0.4602 (0.2933-0.6271)	0.082	-0.486	0.938
DNMT1.56.57	62	1.14	1.32	0.37	0.68	0.3231 (0.1975-0.4488)	0.062	-2.835	0.136

Group comparisons are conducted with Brunner-Munzel tests (see 2.6 for details). Multiple testing correction by false discovery rate was conducted and only adjusted *p* values (p_{adj}) were reported. Tests with $p_{adj} < .05$ were marked as bold. CM: childhood maltreatment, SD: standard deviation, LCI: lower 95% confidence interval, UCI: Upper 95% confidence interval, SE: standard error, T: Brunner-Munzel test statistic

Supplementary Table 6: Descriptives and comparisons of individual CpG unit methylation percentages in cord blood immune cells of newborns by maternal history of childhood maltreatment, low cut-off

CpG unit	N	CM_{low-} Mean	CM_{low-} *SD*	CM_{low+} Mean	CM_{low+} *SD*	Estimator (LCI - UCI)	SE	T	p_{adj}
DNMT1.1.2	106	2.19	1.17	2.00	1.03	0.4672 (0.3597-0.5748)	0.054	-0.604	0.861
DNMT1.3.4	108	0.75	0.72	0.60	0.96	0.4061 (0.3051-0.5071)	0.051	-1.845	0.571
DNMT1.5	82	4.59	3.45	4.68	4.59	0.4676 (0.3382-0.5969)	0.065	-0.500	0.861
DNMT1.6	107	2.35	2.15	2.35	2.22	0.4941 (0.384-0.6041)	0.056	-0.107	0.944
DNMT1.7.8	109	3.00	1.38	2.72	1.13	0.4517 (0.3496-0.5537)	0.052	-0.939	0.861
DNMT1.9	100	4.17	2.19	3.94	1.92	0.4639 (0.3499-0.578)	0.058	-0.628	0.861
DNMT1.10	106	4.04	1.73	3.90	1.39	0.4693 (0.3595-0.5791)	0.055	-0.555	0.861
DNMT1.11	108	2.25	1.38	2.50	2.56	0.5031 (0.3943-0.6118)	0.055	0.056	0.955
DNMT1.12	107	1.11	0.99	1.46	1.48	0.5474 (0.4391-0.6557)	0.055	0.869	0.861
DNMT1.13	101	3.96	3.06	3.92	2.97	0.4807 (0.3655-0.5959)	0.058	-0.332	0.861
DNMT1.14.53	107	1.53	2.49	1.92	3.11	0.5339 (0.427-0.6407)	0.054	0.629	0.861
DNMT1.15.32.47	108	4.95	2.45	4.76	2.71	0.4742 (0.3631-0.5853)	0.056	-0.461	0.861
DNMT1.16	107	2.34	1.59	2.39	1.74	0.4771 (0.3668-0.5873)	0.056	-0.412	0.861
DNMT1.17	110	0.61	1.84	0.83	1.85	0.523 (0.4357-0.6103)	0.044	0.522	0.861
DNMT1.18.19.20	110	28.61	10.67	27.00	15.10	0.4528 (0.341-0.5646)	0.056	-0.838	0.861
DNMT1.21.52	109	6.61	9.71	5.98	12.52	0.435 (0.3323-0.5377)	0.052	-1.255	0.861
DNMT1.22.23	108	5.59	3.42	7.06	8.73	0.489 (0.3781-0.5999)	0.056	-0.197	0.901
DNMT1.24.25.26.27	106	3.02	1.84	3.35	4.11	0.4802 (0.369-0.5915)	0.056	-0.353	0.861
DNMT1.28.29.30	99	2.86	1.97	3.25	2.12	0.5466 (0.4337-0.6595)	0.057	0.819	0.861
DNMT1.31	107	0.56	1.62	0.76	1.80	0.5146 (0.4309-0.5982)	0.042	0.346	0.861
DNMT1.33	109	2.82	2.67	5.17	5.28	0.6186 (0.5108-0.7263)	0.054	2.186	0.502
DNMT1.34	99	2.55	1.98	2.33	2.00	0.4708 (0.3558-0.5858)	0.058	-0.504	0.861
DNMT1.35	101	2.49	2.19	2.81	2.26	0.5385 (0.4249-0.6521)	0.057	0.673	0.861
DNMT1.36.37	108	4.91	1.97	4.73	2.67	0.4501 (0.3413-0.559)	0.055	-0.909	0.861
DNMT1.38.39	108	4.11	1.61	4.94	6.16	0.422 (0.3115-0.5326)	0.056	-1.402	0.861
DNMT1.40	101	1.68	1.57	1.83	1.68	0.5189 (0.4069-0.6308)	0.056	0.334	0.861
DNMT1.41	107	6.88	8.54	6.40	8.51	0.484 (0.3725-0.5956)	0.056	-0.284	0.861
DNMT1.42.43	108	3.25	4.04	2.85	1.92	0.4845 (0.3747-0.5944)	0.055	-0.279	0.861
DNMT1.44.45.46	108	11.07	3.76	10.81	6.84	0.4038 (0.2927-0.515)	0.056	-1.717	0.571
DNMT1.48.49.50.51	109	1.96	2.89	3.02	7.80	0.4695 (0.3613-0.5777)	0.054	-0.560	0.861
DNMT1.54.55	105	4.82	2.36	4.00	2.17	0.4037 (0.2954-0.512)	0.055	-1.763	0.571
DNMT1.56.57	106	1.75	1.56	1.22	1.40	0.3764 (0.2746-0.4783)	0.051	-2.407	0.502

Group comparisons are conducted with Brunner-Munzel tests (see 2.6 for details). Multiple testing correction by false discovery rate was conducted and only adjusted *p* values (p_{adj}) were reported. Tests with $p_{adj} < .05$ were marked as bold. CM: childhood maltreatment, SD: standard deviation, LCI: lower 95% confidence interval, UCI: Upper 95% confidence interval, SE: standard error, T: Brunner-Munzel test statistic

Supplementary Table 7: Descriptives and comparisons of individual CpG unit methylation percentages in cord blood immune cells of newborns by maternal history of childhood maltreatment, moderate cut-off

**CpG unit**	**N**	**CM_{mod-}**		**CM_{mod+}**		**Estimator (LCI - UCI)**	**SE**	**T**	**p_{adj}**
		**Mean**	***SD***	**Mean**	***SD***				
DNMT1.1.2	106	2.04	1.12	2.24	1.06	0.5513 (0.4373-0.6653)	0.057	0.899	0.549
DNMT1.3.4	108	0.72	0.83	0.55	0.87	0.4295 (0.3163-0.5427)	0.056	-1.252	0.413
DNMT1.5	82	5.20	4.20	3.42	3.42	0.3685 (0.2387-0.4982)	0.065	-2.032	0.413
DNMT1.6	107	2.19	2.12	2.76	2.29	0.5796 (0.4521-0.7071)	0.063	1.257	0.413
DNMT1.7.8	109	2.90	1.28	2.77	1.25	0.4823 (0.366-0.5985)	0.058	-0.306	0.870
DNMT1.9	100	4.07	2.11	4.04	1.95	0.4931 (0.3667-0.6194)	0.063	-0.110	0.968
DNMT1.10	106	3.96	1.62	4.00	1.44	0.5082 (0.383-0.6335)	0.062	0.132	0.968
DNMT1.11	108	2.21	1.34	2.80	3.18	0.5342 (0.4067-0.6617)	0.063	0.540	0.768
DNMT1.12	107	1.08	1.03	1.83	1.63	0.6291 (0.5011-0.757)	0.063	2.040	0.413
DNMT1.13	101	3.70	2.83	4.57	3.39	0.57 (0.4513-0.6886)	0.059	1.177	0.413
DNMT1.14.53	107	1.90	3.06	1.15	1.66	0.4275 (0.3083-0.5468)	0.059	-1.222	0.413
DNMT1.15.32.47	108	5.03	2.54	4.43	2.64	0.412 (0.2871-0.5368)	0.062	-1.416	0.413
DNMT1.16	107	2.44	1.60	2.17	1.81	0.4193 (0.2956-0.543)	0.062	-1.311	0.413
DNMT1.17	110	0.55	1.61	1.17	2.32	0.5471 (0.4406-0.6536)	0.053	0.891	0.549
DNMT1.18.19.20	110	28.25	12.17	26.73	15.04	0.4948 (0.3601-0.6295)	0.067	-0.078	0.968
DNMT1.21.52	109	7.22	11.90	3.90	8.43	0.4274 (0.3185-0.5364)	0.055	-1.331	0.413
DNMT1.22.23	108	5.27	3.49	8.97	10.75	0.5949 (0.4684-0.7213)	0.063	1.509	0.413
DNMT1.24.25.26.27	106	3.35	3.44	2.72	2.12	0.4292 (0.3085-0.55)	0.060	-1.174	0.413
DNMT1.28.29.30	99	2.94	2.12	3.32	1.83	0.591 (0.4718-0.7103)	0.060	1.526	0.413
DNMT1.31	107	0.59	1.57	0.85	2.05	0.5394 (0.4374-0.6413)	0.051	0.779	0.612
DNMT1.33	109	3.70	3.77	4.60	5.40	0.5283 (0.4012-0.6553)	0.063	0.447	0.808
DNMT1.34	99	2.72	2.06	1.75	1.62	0.3654 (0.2467-0.4842)	0.059	-2.269	0.413
DNMT1.35	101	2.75	2.35	2.35	1.77	0.4677 (0.345-0.5904)	0.061	-0.527	0.768
DNMT1.36.37	108	5.00	2.29	4.37	2.36	0.4173 (0.2971-0.5375)	0.060	-1.378	0.413
DNMT1.38.39	108	4.80	4.91	3.72	2.63	0.4035 (0.27-0.537)	0.066	-1.462	0.413
DNMT1.40	101	1.88	1.68	1.43	1.40	0.4163 (0.2936-0.5391)	0.061	-1.367	0.413
DNMT1.41	107	7.09	9.14	5.37	6.11	0.4743 (0.3469-0.6017)	0.063	-0.405	0.814
DNMT1.42.43	108	3.28	3.60	2.47	1.61	0.4077 (0.2882-0.5272)	0.060	-1.548	0.413
DNMT1.44.45.46	108	10.94	5.49	10.97	5.39	0.4974 (0.3689-0.6259)	0.064	-0.041	0.968
DNMT1.48.49.50.51	109	2.65	6.47	2.03	3.60	0.4376 (0.3065-0.5687)	0.065	-0.962	0.547
DNMT1.54.55	105	4.72	2.42	3.67	1.73	0.3939 (0.2781-0.5096)	0.058	-1.834	0.413
DNMT1.56.57	106	1.59	1.45	1.25	1.65	0.3871 (0.268-0.5063)	0.059	-1.906	0.413

Group comparisons are conducted with Brunner-Munzel tests (see 2.6 for details). Multiple testing correction by false discovery rate was conducted and only adjusted p values (p_{adj}) were reported. Tests with $p_{adj} < .05$ were marked as bold. CM: childhood maltreatment, SD: standard deviation, LCI: lower 95% confidence interval, UCI: Upper 95% confidence interval, SE: standard error, T: Brunner-Munzel test statistic

Supplementary Table 8: Correlations of mother-newborn levels of individual CpG unit methylation percentages in immune cells and buccal cells

CpG unit	Immune cells			Buccal cells		
	N	r_s	p_{adj}	N	r_s	p_{adj}
DNMT1.1.2	99	-.240	0.054	67	-.027	0.963
**DNMT1.3.4**	**100**	**.256**	**0.036**	67	-.020	0.963
DNMT1.5	68	.104	0.625	38	-.029	0.963
DNMT1.6	97	-.073	0.666	64	.083	0.797
DNMT1.7.8	100	.077	0.650	67	-.131	0.717
DNMT1.9	92	.172	0.213	67	.070	0.850
DNMT1.10	98	.012	0.929	67	.053	0.906
DNMT1.11	100	.139	0.318	67	-.207	0.412
**DNMT1.12**	**99**	**.401**	**< .01**	65	.189	0.451
DNMT1.13	94	.060	0.676	59	-.136	0.717
**DNMT1.14.53**	**99**	**.278**	**0.015**	56	.275	0.364
DNMT1.15.32.47	99	.014	0.929	58	.062	0.906
DNMT1.16	99	.09	0.068	58	-.230	0.412
**DNMT1.17**	**102**	**.479**	**< .01**	59	-.043	0.961
**DNMT1.18.19.20**	**102**	**.462**	**< .01**	57	-.007	0.992
**DNMT1.21.52**	**95**	**.369**	**< .01**	49	.264	0.412
DNMT1.22.23	97	.153	0.268	53	-.274	0.364
DNMT1.24.25.26.27	95	.064	0.676	54	-.013	0.987
DNMT1.28.29.30	90	-.108	0.556	57	-.127	0.717
**DNMT1.31**	**99**	**.628**	**< .01**	57	.374	0.124
DNMT1.33	97	.058	0.676	N/A	N/A	N/A
DNMT1.34	84	.068	0.676	53	.033	0.963
DNMT1.35	88	.104	0.556	49	.295	0.364
DNMT1.36.37	98	-.043	0.770	58	-.001	0.992
DNMT1.38.39	100	.200	0.123	59	-.166	0.648
DNMT1.40	94	.172	0.213	59	-.091	0.797
DNMT1.41	99	-.022	0.917	58	-.130	0.717
DNMT1.42.43	97	.009	0.929	59	-.205	0.451
**DNMT1.44.45.46**	**100**	**.382**	**< .01**	59	-.094	0.797
**DNMT1.48.49.50.51**	**100**	**.329**	**< .01**	56	.141	0.717
DNMT1.54.55	95	.086	0.625	59	.114	0.748
DNMT1.56.57	98	.177	0.199	59	.109	0.963

Spearman correlations were conducted (see 2.6 for details). Multiple testing correction by false discovery rate was conducted and only adjusted *p* values (p_{adj}) were reported. Tests with $p_{adj} < .05$ were marked as bold. N/A: not available

Supplementary Table 9: Descriptives and comparisons of individual CpG unit methylation percentages in buccal cells of newborns by maternal history of childhood maltreatment, low cut-off

CpG unit	N	CM_{low-}		CM_{low+}		Estimator (LCI - UCI)	SE	T	p_{adj}
		Mean	*SD*	Mean	*SD*				
DNMT1.1.2	67	1.51	0.74	1.75	0.76	0.5848 (0.4578-0.7118)	0.064	1.334	0.784
DNMT1.3.4	67	0.91	0.66	0.97	0.54	0.5366 (0.4336-0.6396)	0.052	0.710	0.846
DNMT1.5	52	3.85	3.12	4.12	3.46	0.5133 (0.3493-0.6774)	0.082	0.163	0.938
DNMT1.6	66	0.97	1.60	0.75	0.80	0.5331 (0.3997-0.6665)	0.067	0.496	0.846
DNMT1.7.8	67	3.40	1.06	3.38	1.13	0.4705 (0.3387-0.6024)	0.066	-0.446	0.846
DNMT1.9	67	2.80	1.35	2.78	1.24	0.5134 (0.3751-0.6517)	0.069	0.194	0.938
DNMT1.10	67	5.69	1.11	5.78	1.45	0.5665 (0.4333-0.6998)	0.067	0.997	0.814
DNMT1.11	67	2.80	1.02	2.59	1.16	0.4402 (0.303-0.5774)	0.069	-0.873	0.846
DNMT1.12	66	3.85	2.68	3.00	1.87	0.4017 (0.2641-0.5392)	0.069	-1.429	0.784
DNMT1.13	65	7.46	5.24	6.03	3.83	0.4657 (0.32-0.6115)	0.073	-0.470	0.846
DNMT1.14	63	0.71	1.61	1.72	2.53	0.5953 (0.4707-0.7199)	0.062	1.537	0.784
DNMT1.15	64	5.00	2.71	5.03	3.19	0.469 (0.3207-0.6172)	0.074	-0.419	0.846
DNMT1.16	64	1.56	1.52	1.47	2.74	0.424 (0.2866-0.5614)	0.069	-1.106	0.814
DNMT1.17	65	0.20	0.68	0.00	0.00	0.4571 (0.4084-0.5059)	0.024	-1.785	0.784
DNMT1.18.19.20	64	38.26	12.08	42.17	13.05	0.573 (0.4272-0.7188)	0.073	1.002	0.814
DNMT1.21	62	30.18	38.42	34.75	40.55	0.4669 (0.3138-0.62)	0.076	-0.435	0.846
DNMT1.22.23	59	5.82	3.19	7.12	4.30	0.5752 (0.4232-0.7271)	0.076	0.992	0.814
DNMT1.24.25.26.27	62	4.37	2.53	4.30	2.22	0.5169 (0.3692-0.6647)	0.074	0.229	0.938
DNMT1.28.29.30	64	4.35	2.83	4.30	1.84	0.6029 (0.4598-0.7461)	0.072	1.438	0.784
DNMT1.31	64	0.03	0.17	0.03	0.18	0.502 (0.4575-0.5464)	0.022	0.088	0.957
DNMT1.32	64	5.00	2.71	5.03	3.19	0.469 (0.3207-0.6172)	0.074	-0.419	0.846
DNMT1.34	61	4.33	4.07	2.64	2.18	0.3939 (0.2501-0.5378)	0.072	-1.476	0.784
DNMT1.35	56	4.45	4.22	4.76	2.83	0.5974 (0.4462-0.7486)	0.075	1.294	0.784
DNMT1.36.37	64	3.85	1.94	3.70	2.28	0.4676 (0.32-0.6153)	0.074	-0.439	0.846
DNMT1.38.39	65	8.03	7.91	9.37	6.44	0.5981 (0.4568-0.7394)	0.071	1.387	0.784
DNMT1.40	65	2.23	3.37	1.67	2.44	0.49 (0.3526-0.6274)	0.069	-0.146	0.938
DNMT1.41	64	59.09	33.67	53.66	36.45	0.4591 (0.3107-0.6075)	0.074	-0.552	0.846
DNMT1.42.43	65	4.91	4.02	5.13	4.44	0.5167 (0.3705-0.6628)	0.073	0.228	0.938
DNMT1.44.45.46	65	16.46	9.66	18.60	9.49	0.5871 (0.4451-0.7292)	0.071	1.226	0.787
DNMT1.47	64	5.00	2.71	5.03	3.19	0.469 (0.3207-0.6172)	0.074	-0.419	0.846
DNMT1.48.49.50.51	63	2.29	3.23	2.31	3.15	0.536 (0.3931-0.6789)	0.072	0.504	0.846
DNMT1.52	62	30.18	38.42	34.75	40.55	0.4669 (0.3138-0.62)	0.076	-0.435	0.846
DNMT1.53	63	0.71	1.61	1.72	2.53	0.5953 (0.4707-0.7199)	0.062	1.537	0.784
DNMT1.54.55	65	4.14	2.20	4.13	2.16	0.4976 (0.3528-0.6425)	0.073	-0.033	0.974
DNMT1.56.57	65	1.69	4.19	1.10	1.47	0.4671 (0.3278-0.6065)	0.070	-0.472	0.846

Group comparisons are conducted with Brunner-Munzel tests (see 2.6 for details). Multiple testing correction by false discovery rate was conducted and only adjusted *p* values (p_{adj}) were reported. Tests with $p_{adj} < .05$ were marked as bold. CM: childhood maltreatment, SD: standard deviation, LCI: lower 95% confidence interval, UCI: Upper 95% confidence interval, SE: standard error, T: Brunner-Munzel test statistic

Supplementary Table 10: Descriptives and comparisons of individual CpG unit methylation percentages in buccal cells of newborns by maternal history of childhood maltreatment, moderate cut-off

CpG unit	N	CM_{mod-}		CM_{mod+}		Estimator (LCI - UCI)	SE	T	p_{adj}
		Mean	*SD*	Mean	*SD*				
DNMT1.1.2	67	1.62	0.75	1.64	0.79	0.4919 (0.3526-0.6313)	0.069	-0.117	0.939
DNMT1.3.4	67	0.96	0.67	0.91	0.43	0.5091 (0.4031-0.6151)	0.053	0.172	0.939
DNMT1.5	52	3.94	3.34	4.06	3.19	0.5134 (0.3417-0.6852)	0.085	0.159	0.939
DNMT1.6	66	0.93	1.45	0.73	0.83	0.5103 (0.3734-0.6472)	0.068	0.152	0.939
DNMT1.7.8	67	3.47	1.01	3.23	1.23	0.3924 (0.2586-0.5263)	0.067	-1.617	0.939
DNMT1.9	67	2.80	1.29	2.77	1.31	0.5061 (0.3476-0.6645)	0.078	0.078	0.939
DNMT1.10	67	5.67	1.41	5.86	0.94	0.5475 (0.4044-0.6906)	0.071	0.669	0.939
DNMT1.11	67	2.78	1.13	2.55	1.01	0.4258 (0.2808-0.5707)	0.072	-1.033	0.939
DNMT1.12	66	3.75	2.71	2.82	1.14	0.4298 (0.2911-0.5684)	0.069	-1.014	0.939
DNMT1.13	65	7.00	5.05	6.35	3.70	0.4872 (0.3372-0.6373)	0.075	-0.171	0.939
DNMT1.14	63	1.14	2.11	1.26	2.23	0.5215 (0.382-0.6611)	0.069	0.314	0.939
DNMT1.15	64	4.93	2.61	5.20	3.56	0.4818 (0.3108-0.6528)	0.084	-0.217	0.939
DNMT1.16	64	1.30	1.47	2.00	3.20	0.5653 (0.4209-0.7098)	0.072	0.910	0.939
DNMT1.17	65	0.16	0.60	0.00	0.00	0.4667 (0.4288-0.5046)	0.019	-1.773	0.939
DNMT1.18.19.20	64	40.34	12.61	39.55	12.87	0.4636 (0.3021-0.6251)	0.080	-0.456	0.939
DNMT1.21	62	34.80	40.19	26.00	36.79	0.4066 (0.2415-0.5716)	0.081	-1.156	0.939
DNMT1.22.23	59	6.00	3.26	7.28	4.65	0.586 (0.4115-0.7606)	0.085	1.009	0.939
DNMT1.24.25.26.27	62	4.51	2.60	3.95	1.78	0.47 (0.3159-0.6241)	0.076	-0.393	0.939
DNMT1.28.29.30	64	4.30	2.62	4.40	1.88	0.5943 (0.4433-0.7453)	0.075	1.259	0.939
DNMT1.31	64	0.05	0.21	0.00	0.00	0.4773 (0.4452-0.5093)	0.016	-1.431	0.939
DNMT1.32	64	4.93	2.61	5.20	3.56	0.4818 (0.3108-0.6528)	0.084	-0.217	0.939
DNMT1.34	61	3.74	3.95	3.16	1.80	0.5138 (0.3666-0.661)	0.074	0.188	0.939
DNMT1.35	56	4.74	4.02	4.24	2.63	0.509 (0.3512-0.6669)	0.078	0.115	0.939
DNMT1.36.37	64	3.59	1.98	4.20	2.31	0.5574 (0.3898-0.725)	0.082	0.699	0.939
DNMT1.38.39	65	8.78	7.86	8.35	5.82	0.5067 (0.3532-0.6601)	0.076	0.088	0.939
DNMT1.40	65	1.84	3.07	2.25	2.79	0.5822 (0.4341-0.7304)	0.073	1.122	0.939
DNMT1.41	64	59.91	34.86	49.40	34.36	0.4045 (0.2502-0.5589)	0.076	-1.250	0.939
DNMT1.42.43	65	4.84	4.19	5.40	4.26	0.5711 (0.4231-0.7191)	0.074	0.965	0.939
DNMT1.44.45.46	65	17.78	10.16	16.70	8.27	0.4889 (0.3344-0.6434)	0.077	-0.145	0.939
DNMT1.47	64	4.93	2.61	5.20	3.56	0.4818 (0.3108-0.6528)	0.084	-0.217	0.939
DNMT1.48.49.50.51	63	2.67	3.54	1.50	2.04	0.4448 (0.3013-0.5883)	0.072	-0.772	0.939
DNMT1.52	62	34.80	40.19	26.00	36.79	0.4066 (0.2415-0.5716)	0.081	-1.156	0.939
DNMT1.53	63	1.14	2.11	1.26	2.23	0.5215 (0.382-0.6611)	0.069	0.314	0.939
DNMT1.54.55	65	3.98	2.02	4.50	2.48	0.5622 (0.3914-0.733)	0.083	0.746	0.939
DNMT1.56.57	65	1.47	3.75	1.30	1.53	0.5506 (0.3991-0.702)	0.075	0.677	0.939

Group comparisons are conducted with Brunner-Munzel tests (see 2.6 for details). Multiple testing correction by false discovery rate was conducted and only adjusted *p* values (p_{adj}) were reported. Tests with $p_{adj} < .05$ were marked as bold. CM: childhood maltreatment, SD: standard deviation, LCI: lower 95% confidence interval, UCI: Upper 95% confidence interval, SE: standard error, T: Brunner-Munzel test statistic

Supplementary references

1. Dreos R, Ambrosini G, Périer RC, Bucher P. The Eukaryotic Promoter Database: expansion of EPDnew and new promoter analysis tools. *Nucleic Acids Research* (2015) 43:D92–D96. doi: 10.1093/nar/gku1111

2. Abugessaisa I, Noguchi S, Hasegawa A, Kondo A, Kawaji H, Carninci P, Kasukawa T. refTSS: A Reference Data Set for Human and Mouse Transcription Start Sites. *Journal of Molecular Biology* (2019) 431:2407–2422. doi: 10.1016/j.jmb.2019.04.045

3. ENCODE Project Consortium. An integrated encyclopedia of DNA elements in the human genome. *Nature* (2012) 489:57–74. doi: 10.1038/nature11247

4. Karagkouni D, Paraskevopoulou MD, Chatzopoulos S, Vlachos IS, Tastsoglou S, Kanellos I, Papadimitriou D, Kavakiotis I, Maniou S, Skoufos G, et al. DIANA-TarBase v8: a decade-long collection of experimentally supported miRNA–gene interactions. *Nucleic Acids Research* (2018) 46:D239–D245. doi: 10.1093/nar/gkx1141

5. NCBI Resource Coordinators. Database resources of the National Center for Biotechnology Information. *Nucleic Acids Res* (2016) 44:D7-19. doi: 10.1093/nar/gkv1290
